# Dual Targeting of Mutant p53 and SNRPD2 via Engineered Exosomes Modulates Alternative Splicing to Suppress Ovarian Cancer

**DOI:** 10.1002/advs.202513369

**Published:** 2026-01-20

**Authors:** Wei Zhao, Qian Hao, Yu Gan, Jing Tong, Xiaodan Chen, Shuran Tan, Ruiwen Ruan, Yingdan Huang, Mingming Cao, Jun Deng, Tao Han, Getao Shi, Bo Gao, Yu Zhang, Xiang Zhou

**Affiliations:** ^1^ Department of Gynecology Xiangya Hospital Central South University Changsha China; ^2^ Gynecological Oncology Research and Engineering Center of Hunan Province Changsha China; ^3^ National Clinical Research Center for Geriatric Disorders Xiangya Hospital Central South University Changsha China; ^4^ Fudan University Shanghai Cancer Center and Institutes of Biomedical Sciences Fudan University Shanghai China; ^5^ Department of Oncology Shanghai Medical College Fudan University Shanghai China; ^6^ Department of Oncology The First Affiliated Hospital Jiangxi Medical College Nanchang University Nanchang Jiangxi China; ^7^ Jiangxi Key Laboratory For Individual Cancer Therapy Nanchang Jiangxi China; ^8^ Xinxiang Key laboratory For Molecular Oncology Institutes of Health Central Plains Xinxiang Medical University Xinxiang China; ^9^ Umibio Co. Ltd. Shanghai China; ^10^ Key Laboratory of Breast Cancer in Shanghai Department of Breast Surgery Fudan University Shanghai Cancer Center Fudan University Shanghai China

**Keywords:** alternative splicing, engineered exosomes, p53, SNRPD2, targeted therapy

## Abstract

Mutation of the tumor suppressor gene *TP53* promotes ovarian cancer progression and therapeutic resistance. Whether mutant p53 (mtp53) regulates alternative splicing and how this regulation can be exploited for cancer therapy remain unclear. Here, small nuclear ribonucleoprotein D2 polypeptide (SNRPD2) as a binding partner of mtp53 is identified. SNRPD2 is highly expressed in ovarian cancer and associated with an unfavorable prognosis. The overexpression of SNRPD2 promotes, whereas its depletion inhibits, the growth and migration of ovarian cancer cells. Mechanistically, mtp53 cooperates with SNRPD2 to facilitate the assembly of the Sm/SMN protein complex, an essential component of the spliceosome, modulating alternative splicing of pre‐mRNAs. Specifically, the co‐depletion of mtp53 and SNRPD2 reduces the level of OTUD3 oncogenic transcripts while increasing its tumor suppressor counterparts through an exon‐skipping event. Moreover, therapeutic engineered exosomes are developed with their surfaces decorated with iRGD and their interiors loaded with siRNAs targeting mtp53 and SNRPD2. These exosomes effectively suppress the growth of ovarian cancer cells and enhance their sensitivity to chemotherapy in vivo. Collectively, this study uncovers that mtp53 and SNRPD2 cooperatively regulate alternative splicing to drive ovarian cancer progression, and co‐targeting these two molecules via engineered exosomes represents a potential therapeutic strategy for ovarian cancer.

## Graphical abstract

Mutant p53 drives oncogenic splicing to promote the progression of ovarian cancer by partnering with the spliceosome factor SNRPD2. Therefore, it is engineered iRGD‐exosomes to co‐deliver siRNAs against both targets. This approach restored tumor‐suppressive mRNA isoforms, effectively enhanced sensitivity to cisplatin, and ultimately blocked tumor progression.

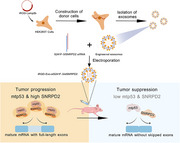

## 1 Introduction

Ovarian cancer, characterized by frequent relapse and inherent chemotherapy resistance, ranks among the most lethal gynecological malignancies [1]. Despite the continuous development of novel therapeutic strategies, clinical management remains severely challenged by the high relapse rate and chemotherapy resistance [2, 3, 4]. *TP53* mutations represent a cornerstone molecular alteration in ovarian cancer (OC), particularly in high‐grade serous ovarian carcinoma (HGSOC), with profound clinical and biological implications [5]. *TP53* mutations are detected in over 96% of HGSOC cases [6]. The mechanisms underlying mutant p53 (mtp53)‐mediated tumor development involves loss‐of‐function (LOF), dominant‐negative effects, and gain‐of‐function (GOF) [7, 8, 9]. GOF refers to the situation where mtp53 not only loses the tumor suppressive functions but also acquires oncogenic functions that actively drive cancer progression and resistance to therapy [10, 11, 12]. Deciphering the pleiotropic functions of mtp53 may provide both fundamental insights into HGSOC biology and actionable targets to overcome relapse after primary therapy [13, 14, 15, 16].

Alternative splicing, a rigorously regulated post‐transcriptional mechanism, enables a single gene to generate multiple mRNA isoforms, significantly expanding proteomic diversity [17, 18]. Its dysregulation is a pervasive driver of cancer, contributing to tumor initiation, progression, metastasis, and therapeutic resistance, offering novel biomarkers and precision therapy avenues [19, 20]. p53 mutant isoforms, such as mtp53‐R175H, have been implicated in the regulation of RNA splicing to generate GTPase‐activating protein (GAP) isoforms, which enhances the activity of KRAS to promote the growth of pancreatic ductal adenocarcinoma (PDAC) [21]. However, modulating alternative splicing by mtp53 in ovarian cancer has yet to be determined.

Small nuclear ribonucleoprotein D2 polypeptide (SNRPD2, also known as SmD2) is a component of the core Sm protein complex, consisting of seven Sm proteins, including SmB/B', SmD1, SmD2, SmD3, SmE, SmF, and SmG [22, 23, 24]. These proteins form a ring‐like structure that associates with SMN proteins and small nuclear RNAs (snRNAs), thus serving as a scaffold for the assembly of small nuclear ribonucleoprotein particles (snRNPs). This assembly is crucial for the formation of the active spliceosome, a key molecular machine in pre‐mRNA splicing [17, 24]. SNRPD2 dysregulation is associated with aberrant mRNA isoform generation, contributing to various diseases, including cancer [23, 25, 26, 27, 28].

In this study, we identify SNRPD2 as an interacting partner of mtp53 in ovarian cancer using proteomic analysis combined with co‐immunoprecipitation (co‐IP) validation. Our results demonstrate that SNRPD2 specifically interacts with mtp53 but not wild type p53 (wtp53). This interaction facilitates the assembly of the spliceosome, as the depletion of mtp53 impairs the interaction between SNRPD2 and SMN. Transcriptomic analyses reveal that the knockdown of either mtp53 or SNRPD2 can result in a splicing switch of the same subset of genes, including *OTUD3*, *EAF2*, *GSTO2*, and *FOCAD*. Previous studies have identified that OTUD3, a deubiquitinase of the OTU family, plays a role in tumor growth by controlling protein stability [29, 30]. Our results show that the depletion of either mtp53 or SNRPD2 reduces the long‐form, tumor‐promoting transcript of OTUD3 (OTUD3‐L) and increases the short‐form, tumor‐suppressive transcript of OTUD3 (OTUD3‐S). The co‐depletion of mtp53 and SNRPD2 exhibits a synergistic effect in inhibiting the growth of ovarian cancer in vivo. Notably, our study has developed an exosome‐based siRNA delivery system that selectively targets mtp53‐S241F (but not wtp53 or other mtp53s) and SNRPD2. These engineered exosomes can effectively suppress ovarian cancer growth and synergize with cisplatin to enhance therapeutic efficacy in vivo. Collectively, our study uncovers that mtp53 collaborates with SNRPD2 to modulate alternative splicing, and simultaneously targeting both molecules using engineered exosomes could be a potential therapeutic strategy for ovarian cancer.

## 2 Results

### 2.1 Identification of SNRPD2 as a Mutant p53‐binding Protein in Ovarian Cancer

**FIGURE 1:**
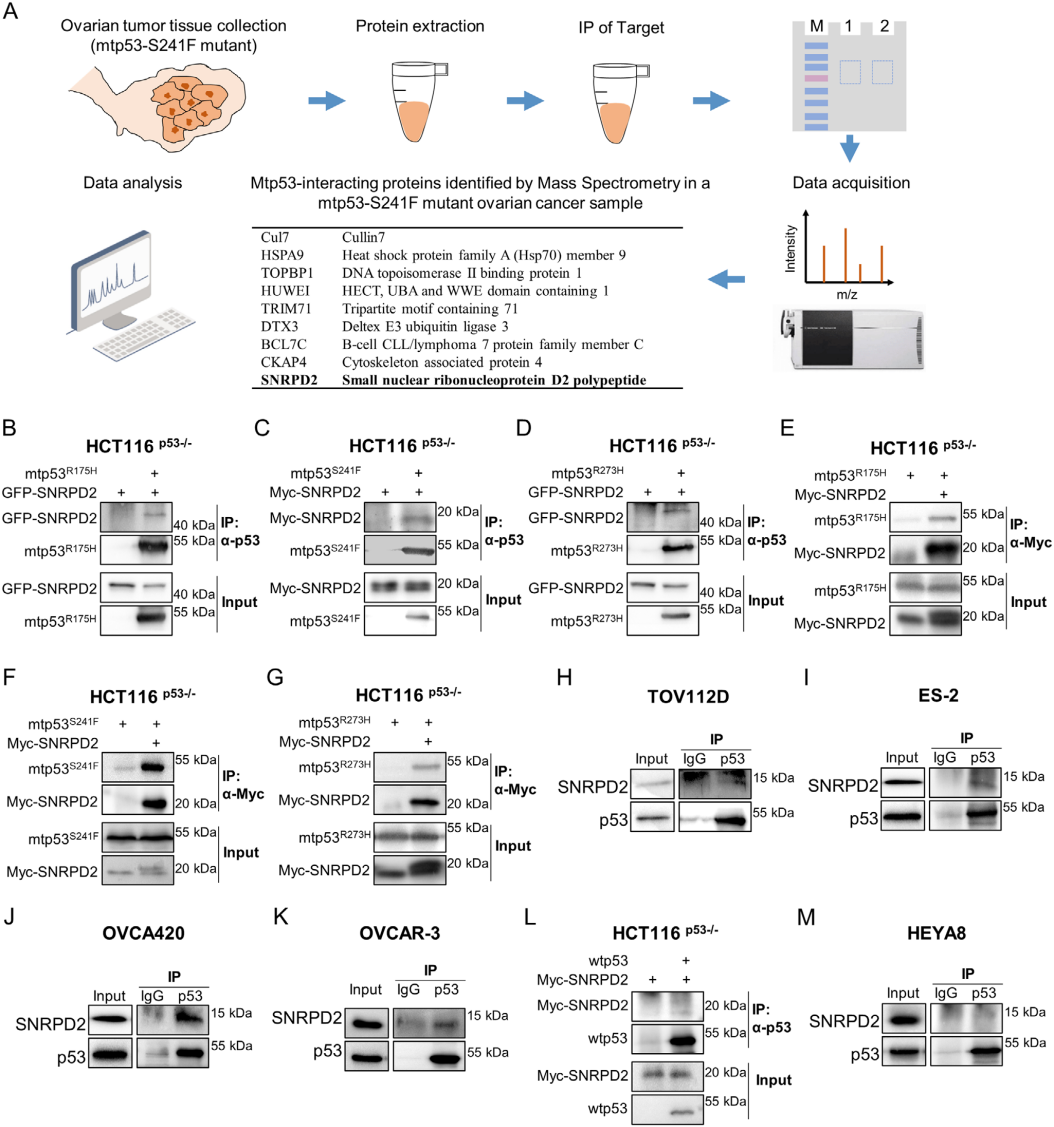
Identification of SNRPD2 as a mutant p53‐binding protein in ovarian cancer. (A) Workflow for immunoprecipitation‐mass spectrometry (IP‐MS) identification of mtp53 binding proteins from a clinical ovarian tumor tissue harboring mtp53‐S241F. (B–G) HCT116^p53‒/‒^ cells were transfected with the indicated plasmids, followed by co‐IP‐IB analysis using antibodies as indicated. (H–K) TOV112D (mtp53‐R175H), ES‐2 (mtp53‐S241F), OVCA420 (mtp53‐R273H), and OVCAR‐3 (mtp53‐R248Q) cells were treated with MG132 (20 µM) for 6 h, followed by co‐IP‐IB analysis. (L) HCT116^p53‒/‒^ cells were transfected with the indicated plasmids, followed by co‐IP‐IB analysis. (M) HEYA8 (wtp53) cells were treated with MG132 (20 µM) for 6 h, followed by co‐IP‐IB analysis.

*TP53*, the most frequently mutated gene in human cancers, is highly mutated in ovarian cancer. To elucidate the mechanism underlying mtp53‐mediated development of ovarian carcinoma, we employed a proteomic approach to identify mtp53‐binding proteins in an ovarian cancer tissue harboring mtp53‐S241F [13] and found that SNRPD2 is one of the binding partners of mtp53 (Figure 1A). This primary screening was reliable, because several known wtp53‐ or mtp53‐interacting proteins, such as Cullins [31], HSPA9 [32], TOPBP1 [33], and HUWE1 [34], were consistently found in the tumor sample. Then, we carried out multiple co‐immunoprecipitation‐immunoblotting (co‐IP‐IB) assays using an anti‐p53 antibody and validated the interaction between exogenous SNRPD2 and various p53 mutants, including mtp53‐R175H, mtp53‐S241F, and mtp53‐R273H (Figure 1B–D). Consistently, reverse co‐IP assays using an anti‐Myc antibody also revealed that Myc‐SNRPD2 bound to mtp53s (Figure 1E–G). We further verified the interactions between endogenous SNRPD2 and mtp53s in TOV112D cells with mtp53‐R175H (Figure 1H), ES‐2 cells with mtp53‐S241F (Figure 1I), OVCA420 cells with mtp53‐R273H (Figure 1J), and OVCAR‐3 cells with mtp53‐R248Q (Figure 1K). Interestingly, no interactions were observed between either exogenously or endogenously expressed SNRPD2 and wtp53 (Figure 1L,M). These interactions were reproducibly observed in two independent experiments (Figure S1A–K). Collectively, these results demonstrate that SNRPD2 binds to mtp53s but not wtp53 in ovarian cancer. However, the precise consequences of this interaction remain to be determined.

### 2.2 SNRPD2 Promotes the Growth and Migration of Ovarian Cancer Cells

**FIGURE 2:**
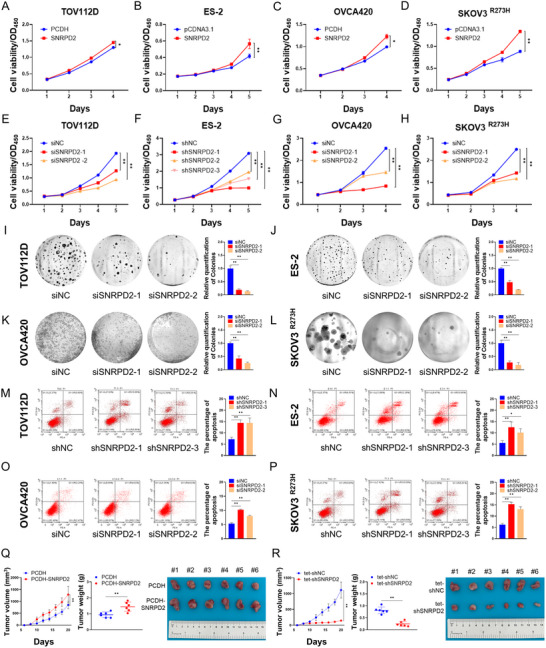
SNRPD2 promotes the progression of ovarian cancer in vitro and in vivo. (A–P) TOV112D, ES‐2, OVCA420, and SKOV3^p53‐R273H^ cells were transfected with the plasmids, siRNAs, or shRNAs as indicated, followed by the cell viability assay (A–H), colony formation assay (I–L), and flow cytometric analysis (M–P). Data are represented as mean ± SD, *n* = 3. * *p* < 0.05, ** *p* < 0.01. (Q,R) The growth rate, weight, and size of xenograft tumors derived from ES‐2 cells stably transfected with the plasmids or shRNAs as indicated. Data in Q and R are represented as mean ± SD, *n* = 6. * *p* < 0.05, ** *p* < 0.01.

To determine the role of SNRPD2 in ovarian cancer, we first performed the CCK‐8 cell viability assay in TOV112D, ES‐2, OVCA420, and SKOV3^p53‐R273H^ cells. The latter is an engineered cell line stably expressing the exogenous mtp53‐R273H [14]. Our results showed that the overexpression of SNRPD2 significantly accelerated ovarian cancer cell proliferation (Figure 2A–D) in these four cell lines. Conversely, the knockdown of SNRPD2 using two or three independent siRNAs or short hairpin RNAs (shRNAs) retarded ovarian cancer cell proliferation (Figure 2E–H). To test whether SNRPD2 affects long‐term cell growth, we conducted the colony formation assay. Our results showed that the overexpression of SNRPD2 enhanced the colony‐forming ability of ovarian cancer cells (Figure S2A,B), whereas the depletion of SNRPD2 markedly inhibited colony formation (Figure 2I–L). In addition, flow cytometric analysis was conducted to test whether SNRPD2 influences apoptosis in ovarian cancer cells. As anticipated, the knockdown of SNRPD2 significantly promoted apoptosis (Figure 2M–P). Furthermore, we established ovarian tumor xenograft models to understand the biological significance of SNRPD2 in the growth of ovarian tumors. ES‐2 cells stably overexpressing SNRPD2 or overexpressing a doxycycline‐inducible shRNA targeting SNRPD2 were subcutaneously transplanted into nude mice. Consistent with our cell‐based results, the overexpression of SNRPD2 significantly increased the tumor growth rate, weight, and size compared with the control group (Figure 2Q). In contrast, doxycycline‐induced SNRPD2 depletion dramatically suppressed the growth of ovarian tumors (Figure 2R).

Next, we examined whether SNRPD2 regulates the migration of ovarian cancer cells. First, the wound‐healing assay was conducted to assess the migratory ability of ovarian cancer cells. Our results revealed that the overexpression of SNRPD2 increased the migration of various ovarian cancer cells (Figure S3A–D), while the depletion of SNRPD2 markedly impeded cell migration (Figure S3E–H). Additionally, the migratory ability of ovarian cancer cells was evaluated by the transwell cell migration assay. Consistently, the overexpression of SNRPD2 enhanced the migration (Figure S3I–K), but the knockdown of SNRPD2 inhibited the migration of ovarian cancer cells (Figure S3L–N). Together, the above results demonstrate that SNRPD2 functions as an oncogenic protein, thereby facilitating the growth and progression of ovarian cancer.

### 2.3 Mutant p53 Collaborates With SNRPD2 to Modulate Splicing Switch

**FIGURE 3:**
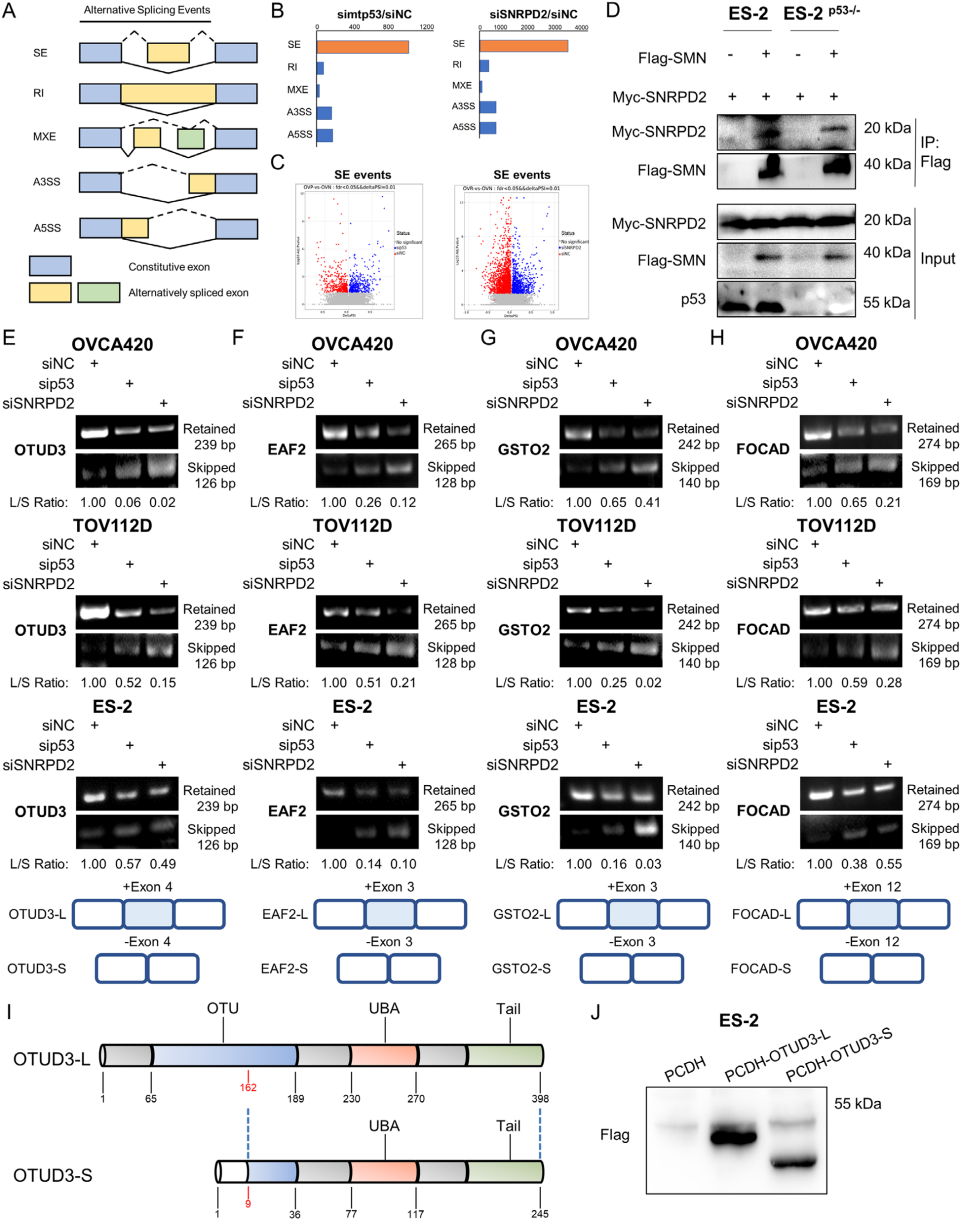
Mutant p53 collaborates with SNRPD2 to modulate splicing switch. (A) An overview of pre‐mRNA splicing events. The blue bars represent constitutive exons adjacent to alternative splicing events. Yellow and green bars represent sequences that are excluded or included depending on the alternative splicing event type. (B,C) OVCA420 cells were transfected with the indicated siRNAs, followed by RNA‐sequencing analysis. Alternative splicing (AS) events are classified into five categories (SE, RI, MXE, A3SS, and A5SS) (B), and these SE events are displayed in volcano plots (C). (D) ES‐2 or ES‐2^p53‒/‒^ (generated by CRISPR/Cas9) cells were transfected with the indicated plasmids, followed by co‐IP‐IB analysis. (E–H) OVCA420, TOV112D, and ES‐2 cells were transfected with the indicated siRNAs, followed by RT‐PCR analysis. ImageJ software was used to quantify the band intensity. (I,J) The schematic (I) and IB validation (J) of OTUD3 splicing isoforms (OTUD3‐L and OTUD3‐S) generated by exon skipping, resulting from the knockdown of p53 and SNRPD2.

Alternative splicing of pre‐mRNAs is a precisely modulated post‐transcriptional process in eukaryotic cells, which occurs in response to environmental stimuli and contributes to RNA, protein, and cell diversity [17, 20, 24]. As an Sm protein, SNRPD2 is considered an alternative splicing regulator. Given that mtp53 interacts with SNRPD2, we were intrigued to explore whether these two molecules co‐regulate the splicing of pre‐mRNAs. We performed RNA‐sequencing (RNA‐seq) analysis of OVCA420 ovarian cancer cells with the knockdown of either mtp53 or SNRPD2 (Figure S4A,B), revealing a subset of differential spliced genes (DSG) that might be co‐regulated by both mtp53 and SNRPD2 (Figure S4A). Analysis of alternative splicing events, with screening criteria of false discovery rate (FDR) < 0.05 and |IncLevelDifference| > 0.01, revealed that p53 or SNRPD2 ablation triggered diverse alternative splicing events. These events fell into five categories: skipped exons (SE), retained introns (RI), mutually exclusive exons (MXE), alternative 3’ splice sites (A3SS), and alternative 5’ splice sites (A5SS) (Figure 3A). In comparison with control groups, our results revealed predominant SE‐type alternative splicing events in response to the depletion of either mtp53 or SNRPD2 (Figure 3B,C). Gene Ontology (GO) enrichment analysis further indicated that these alternative splicing events are involved in various aspects of cancer initiation and progression (Figure S5A). Since the association of SMN with the Sm protein complex is essential for the proper function of the spliceosome [17, 35], we determined whether mtp53 modulates the interaction between SMN and SNRPD2. First, we verified the interaction between SNRPD2 and SMN through co‐IP‐IB assays (Figure S5B). Remarkably, this interaction was impaired upon the depletion of mtp53‐S241F in ES‐2 cells (Figure 3D). These results suggest that mtp53 may facilitate the association of the Sm complex with SMN proteins, thereby influencing the function of the spliceosome (Figure S5C). To provide preliminary mechanistic insight into the potential role of mtp53 in regulating spliceosome assembly, we turned to the well‐characterized interaction between SNRPD1 and SNRPD2—two core components that form the heterodimeric Sm RING and maintain stoichiometric association throughout spliceosome assembly and cycling [36, 37, 38]. Our results revealed that mtp53 depletion did not alter the binding of SNRPD1 to SNRPD2, indicating that the mtp53‐SNRPD2 interaction does not affect the canonical Sm RING core assembly (Figure S6A). However, we cannot exclude the possibility that mtp53 might influence spliceosome assembly through SNRPD2‐independent mechanisms. Finally, we validated several SE‐type alternative splicing events that were potentially regulated by both mtp53 and SNRPD2. Our results showed that the knockdown of either mtp53 or SNRPD2 led to a reduction in the levels of the long transcripts of OTUD3, EAF2, GSTO2, and FOCAD, but increased the production of their corresponding short transcripts lacking an exon (Figure 3E–H). These findings were reproducibly observed in two independent experiments (Figure S6B–F). We selected OTUD3 for functional follow‐up because siRNA‐mediated knockdown of OTUD3 in ES‐2 and TOV112D cells produced the most pronounced effect on cell viability compared with EAF2, FOCAD and GSTO2 (Figure S7A–D).

OTUD3 is an OTU domain‐containing deubiquitinase that regulates various signaling pathways. The skipped exon 4 in OTUD3 is located within its OTU domain, which provides deubiquitinating enzyme (DUB) activity. This activity is crucial for removing ubiquitin from target proteins, thereby regulating their stability and functions [39, 40]. Interestingly, this exon skipping event caused a frameshift mutation and thus an alternative translation initiation site, resulting in a short form of OTUD3 (OTUD3‐S) that lacks the N‐terminal domain and most of the OTU domain (Figure 3I). The expression of both splicing variants, OTUD3‐L and ‐S, was further validated through the IB analysis (Figure 3J).

### 2.4 OTUD3‐L Promotes Whereas OTUD3‐S Suppresses Ovarian Cancer Cell Proliferation and Migration

**FIGURE 4:**
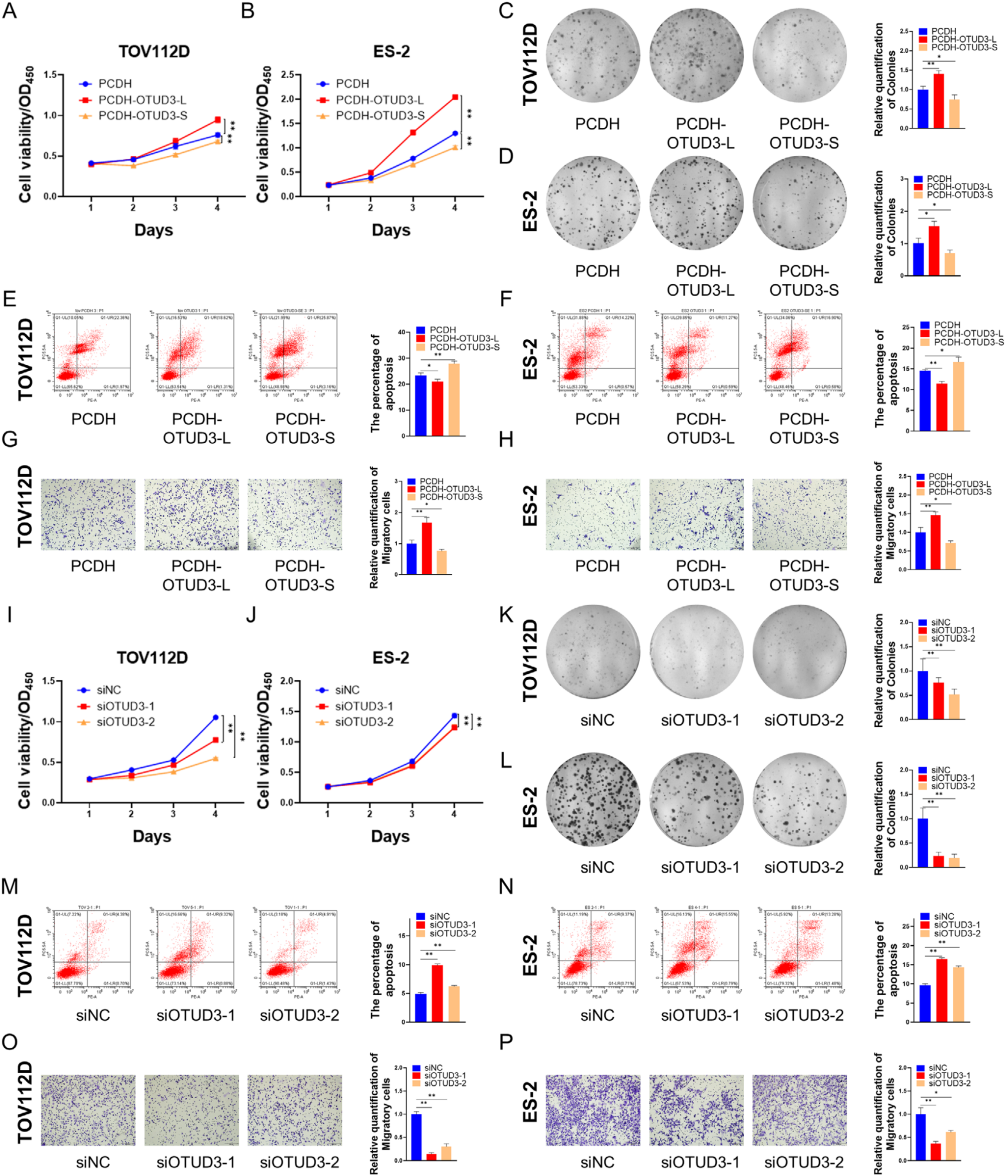
OTUD3‐L promotes whereas OTUD3‐S suppresses ovarian cancer cell proliferation and migration. (A–H) TOV112D and ES‐2 cells were transfected with the plasmids as indicated, followed by the cell viability assay (A,B), colony formation assay (C,D), flow cytometric analysis (E,F), and cell migration assay (G,H). (I–P) TOV112D and ES‐2 cells were transfected with the indicated siRNAs, followed by the cell viability assay (I,J), colony formation assay (K,L), flow cytometric analysis (M,N), and cell migration assay (O,P). Data are represented as mean ± SD, *n* = 3. * *p* < 0.05, ** *p* < 0.01. Scale bars in G, H, O, and P: 100 µm.

Taking OTUD3 as an example, we next investigated how mtp53/SNRPD2‐regulated alternative splicing modulates ovarian cancer cell proliferation. To explore the effects of different OTUD3 isoforms on ovarian cancer cells, we generated TOV112D and ES‐2 cell lines that stably overexpress OTUD3‐L or OTUD3‐S. The cell viability assay and colony formation assay revealed that the overexpression of OTUD3‐L promoted the proliferation and colony formation of ovarian cancer cells (Figure 4A–D). In contrast, OTUD3‐S overexpression inhibited their proliferative capacity and colony‐forming ability (Figure 4A–D). Consistently, OTUD3‐L reduced whereas OTUD3‐S increased apoptosis in ovarian cancer cells through flow cytometric analysis (Figure 4E,F). Our results also showed that OTUD3‐L enhanced the migration of ovarian cancer cells, whereas OTUD3‐S exerted the opposite effect (Figure 4G,H). These results demonstrate that OTUD3‐L functions as an oncogenic protein, whereas OTUD3‐S serves as a tumor suppressor to inhibit the progression of ovarian cancer. We also determined the role of OTUD3 in ovarian cancer by knocking down its expression. Since OTUD3‐L is the dominant isoform in ovarian cancer cells (Figure 3E) and it was unfeasible to design siRNAs specific to OTUD3‐S, we evaluated the function of endogenous OTUD3 using two independent siRNAs technically targeting both OTUD3‐L and ‐S. Our results revealed that the knockdown of OTUD3 (mostly OTUD3‐L) inhibited the proliferation (Figure 4I,J) and colony formation (Figure 4K,L), enhanced apoptosis (Figure 4M,N), and impeded the migration (Figure 4O,P) of ovarian cancer cells. Collectively, these results indicate that the depletion of either mtp53 or SNRPD2 represses the levels of oncogenic transcripts while also elevating the expression of tumor suppressor transcripts, thereby inhibiting the progression of ovarian cancer.

### 2.5 Depletion of both Mutant p53 and SNRPD2 Synergistically Suppresses Ovarian Cancer

Since mtp53 and SNRPD2 coordinately regulate alternative splicing, we investigated whether they promote the growth of ovarian cancer in cooperation using p53‐null ovarian cancer cell lines, ES‐2^p53−/−^ (generated by CRISPR/Cas9) and SKOV3. The overexpression of either mtp53 or SNRPD2 significantly stimulated ovarian cancer cell proliferation (Figure S8A,B), colony formation (Figure S8C,D), and migration (Figure S8E,F) while inhibiting apoptosis (Figure S8G,H). Notably, the simultaneous overexpression of mtp53 and SNRPD2 exerted a more profound oncogenic effect in promoting the progression of ovarian cancer cells (Figure S8A–H). In addition, we tested whether the co‐depletion of both mtp53 and SNRPD2 could more effectively suppress ovarian cancer using ES‐2 and OVCA420 cells. As anticipated, the knockdown of either mtp53 or SNRPD2 significantly suppressed ovarian cancer cell proliferation (Figure S9A,B), colony formation (Figure S9C,D), and migration (Figure S9E,F) while promoting apoptosis (Figure S9G,H). More importantly, the simultaneous depletion of both mtp53 and SNRPD2 led to a more dramatic inhibition of ovarian cancer cell growth and migration (Figure S9A–H). These findings suggest that dual targeting of mtp53 and SNRPD2 may represent a promising therapeutic strategy for ovarian cancer treatment.

### 2.6 Design of siRNAs Specific to Mutant p53 Without Wild‐type p53 Interference

**FIGURE 5:**
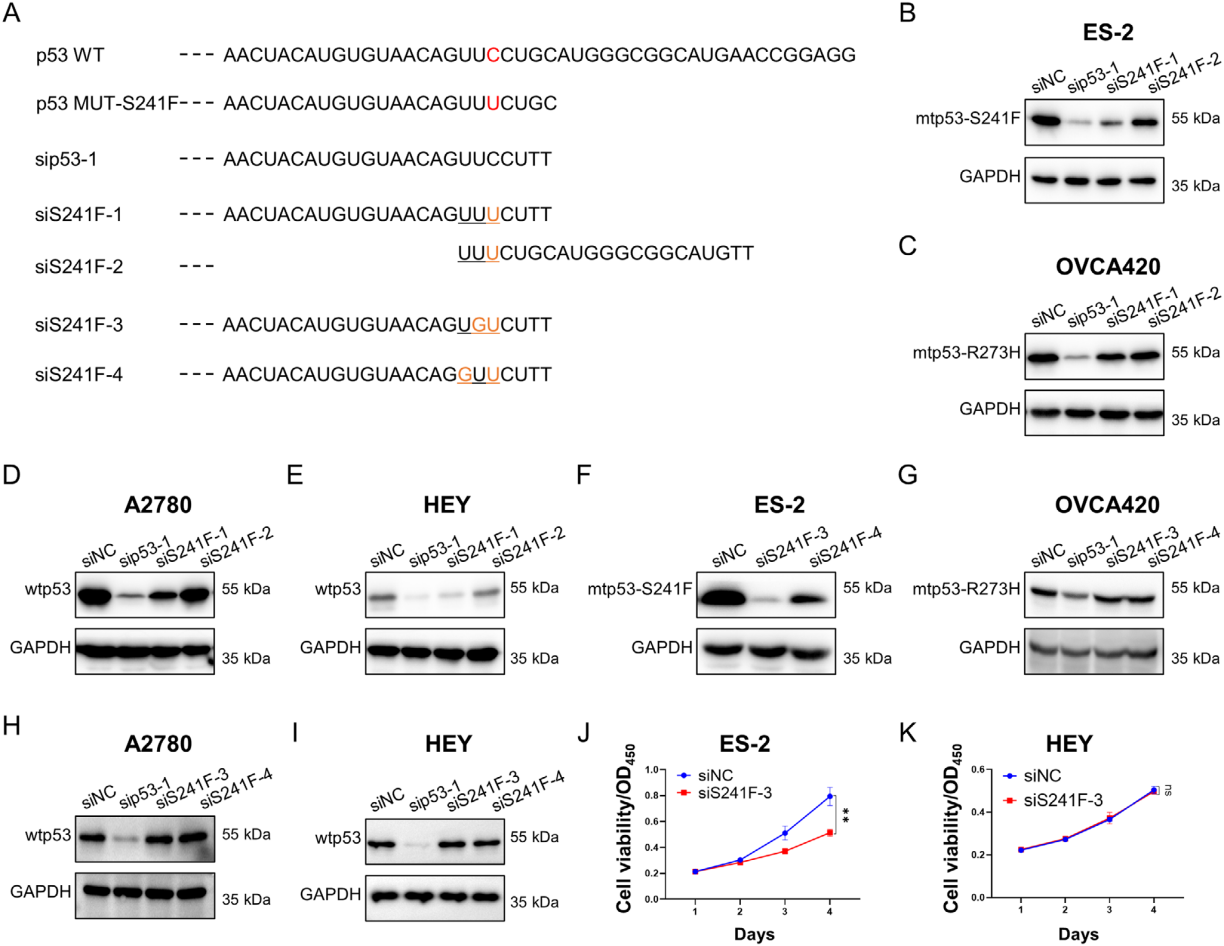
Design of siRNAs specific to mutant p53‐S241F without wild‐type p53 interference. (A) siRNA sequences specifically targeting mtp53‐S241F. The mutated nucleotide residue is highlighted. (B–I) ES‐2 (B and F), OVCA420 (C and G), A2780 (D and H), and HEY (E and I) cells were transfected with indicated siRNAs, followed by IB analysis. (J,K) ES‐2 (J) and HEY (K) cells were transfected with indicated siRNAs, followed by the cell viability assay. Data are represented as mean ± SD, *n* = 3. ** *p* < 0.01, ns, not significant.

The *TP53* gene exhibits an exceptionally high mutation frequency in ovarian carcinoma, wherein GOF mutations (e.g., mtp53‐S241F) acquire oncogenic properties that actively drive tumorigenesis. This underscores the potential of mtp53 as a therapeutic target warranting clinical translation. For instance, arsenic trioxide can restore structural p53 mutations, including R175H, G245S, R249S, and R282W [41], while Rezatapopt specifically reactivates mtp53‐Y220C [42]. Selective inhibition of mtp53 via RNAi technology has been demonstrated as a potential therapeutic strategy for cancer treatment [43, 44]. However, the clinical translation of this approach remains constrained by the absence of efficient RNAi drug delivery vehicles. We sought to develop an RNAi‐based strategy to specifically inhibit mtp53‐S241F in ovarian cancer. First, we designed two siRNAs (siS241F‐1 and ‐2) that were complementary to the mutation site and might potentially be able to selectively silence mtp53‐S241F (Figure 5A). However, IB analysis revealed that siS241F‐1 could still moderately reduce the expression of wtp53, while siS241F‐2 had no impact on either mtp53 or wtp53 expression (Figure 5B–E). To achieve more specific targeting without affecting the expression of wtp53, we designed additional siRNAs (siS241F‐3 and ‐4) featuring one more nucleotide substitution in comparison with siS241F‐1 (Figure 5A). IB analysis demonstrated that siS241F‐3 was able to effectively inhibit the expression of mtp53‐S241F (Figure 5F), while not reducing the expression of wtp53 or other mtp53s (Figure 5G–I). In line with these results, we further showed that siS241F‐3 selectively inhibited the growth of ES‐2 cells bearing mtp53‐S241F, without impacting the growth of cells with wtp53 (Figure 5J,K). The results indicated that siS241F‐3 does not induce uncontrolled proliferation or malignant transformation of normal cells, because it cannot inhibit wtp53. To test whether this mutant‐specific siRNA strategy could be generalized to target other prevalent *TP53* mutations, we designed siRNAs against the mtp53‐R248Q variant (Figure S10A). Indeed, we identified that siR248Q‐2 specifically knocked down mtp53 protein levels in OVCAR‐3 cells harboring mtp53‐R248Q, with no discernible reduction in wtp53 or other mtp53s (Figure S10B–E). Correspondingly, siR248Q‐2 also selectively suppressed the proliferation of OVCAR‐3 cells, while failing to compromise the viability of HEY cells with wtp53 (Figure S10F,G). Therefore, this rationally designed siRNAs are used in the subsequent investigation.

### 2.7 Co‐targeting Mutant p53 and SNRPD2 via iRGD‐Decorated Exosomes Suppresses Ovarian Cancer

**FIGURE 6:**
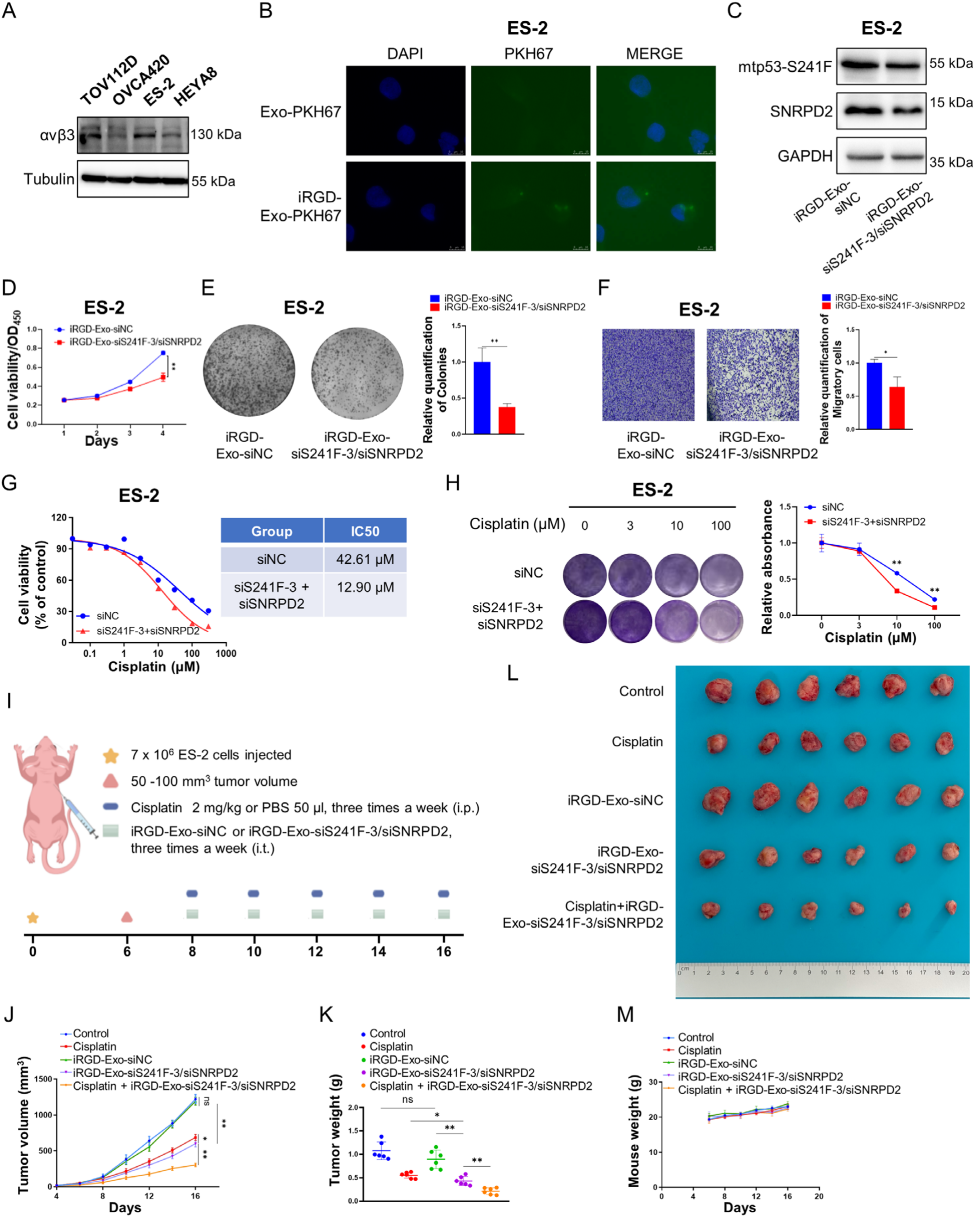
Co‐targeting mutant p53‐S241F and SNRPD2 via iRGD‐decorated exosomes suppresses ovarian cancer. (A) IB analysis of the expression levels of the iRGD ligand in TOV112D, OVCA420, ES‐2, and HEYA8 cells, using antibodies as indicated. (B) ES‐2 cells were incubated with PKH67‐labeled exosomes or PKH67‐labeled iRGD‐exosomes for 8 h, followed by fluorescence microscopy analysis. PKH67‐labeled exosomes or PKH67‐labeled iRGD‐exosomes, green; DAPI, blue for nucleus. (C) ES‐2 cells were incubated with the indicated exosomes for 48 h, followed by IB analysis. (D–F) ES‐2 cells were incubated with the indicated exosomes for 24 h, followed by the cell viability assay (D), colony formation assay (E), and cell migration assay (F). (G,H) ES‐2 cells were transfected with the indicated siRNAs, followed by the cell viability assay (G) and colony formation assay (H). Data are represented as mean ± SD, *n* = 3. * *p* < 0.05, ** *p* < 0.01. Scale bars: 10 µm in B, 100 µm in F. (I) Schematic representation of the treatment timeline for the mouse model. (J–M) The growth rate (J), and weight (K), and size (L) of xenograft tumors derived from ES‐2 cells, along with mouse weight (M), were analyzed as described in the methods section. Data in J, K and M are presented as the mean ± SD, *n* = 6. * *p* < 0.05 and ** *p* < 0.01.

Exosomes, acting as intercellular messengers with great natural biocompatibility and low immunogenicity, can be engineered to display specific surface ligands for selective targeting of diseased cells [45, 46, 47]. Our group has previously established an RNAi‐delivery system based on iRGD‐decorated exosomes for the treatment of colorectal cancer [48]. The exosome‐based delivery system protects siRNAs from degradation, improves cell or tissue penetration, and mitigates immune clearance. Here, we investigated whether co‐targeting mtp53‐S241F and SNRPD2 through this delivery system could be a potential therapeutic approach for ovarian cancer. iRGD‐decorated exosomes were produced as previously described [48]. SiRNAs with the 2’‐O‐methyl modification were used for loading into these exosomes. The morphological characteristics of purified exosomes were verified by transmission electron microscopy (TEM) (Figure S11A) and nanoparticle tracking analysis (NTA) (Figure S11B). The expression of exosomal markers TSG101 and CD63 was confirmed, with endoplasmic reticulum calnexin as a reference for comparison (Figure S11C). The expression of the iRGD ligand αvβ3 was validated in multiple ovarian cancer cells (Figure 6A), and iRGD decoration indeed increased the uptake of PKH67‐labeled exosomes by ES‐2 cells (Figure 6B). These results indicated that the exosomes could serve as tumor‐selective delivery vehicles for ovarian carcinoma. The intended siRNAs were introduced into exosomes through electroporation, producing engineered exosomes, namely iRGD‐Exo‐siNC (iRGD‐decorated exosomes loaded with siNC, a non‐targeting scrambled siRNA serving as the negative control) and iRGD‐Exo‐siS241F‐3/siSNRPD2, for the subsequent investigation.

Next, we tested whether these engineered exosomes suppress the growth and migration of ovarian cancer cells. ES‐2 cells were treated with iRGD‐Exo‐siNC or iRGD‐Exo‐siS241F‐3/siSNRPD2, and the expression of mtp53‐S241F and SNRPD2 was analyzed by the IB assay. Our results showed that iRGD‐Exo‐siS241F‐3/siSNRPD2 repressed the expression of both target molecules (Figure 6C). In addition, these exosomes significantly inhibited the proliferation (Figure 6D), colony formation (Figure 6E), and migration (Figure 6F) of ES‐2 cells. These results demonstrate that the engineered exosomes are able to effectively suppress ovarian cancer progression.

Cisplatin‐containing chemotherapy remains the first‐line treatment for epithelial ovarian carcinoma. Mtp53 has been documented to play a significant role in driving chemotherapy resistance, promoting tumor recurrence, and contributing to poor prognosis [10, 11, 12]. Given that OTUD3 inhibits ferroptosis and confers drug resistance by stabilizing the SLC7A11 protein to elevate intracellular GSH levels [29]. Indeed, upregulation of the SLC7A11‐GSH axis drives resistance to various chemotherapeutics, including cisplatin [49]. However, OTUD3‐S lacks most of the OTU domain (Figure 3I) that is required for deubiquitination and stabilization of SLC7A11. Consequently, we speculated that the engineered exosomes sensitized tumors to cisplatin through a dual mechanism: direct inhibition of mtp53 and a potential reduction in SLC7A11 protein stability. As shown in Figure S12A–D, knockdown of either SNRPD2 or OTUD3 in ES‐2 and TOV112D cells reduced SLC7A11 protein levels without affecting its mRNA expression. Consistently, intracellular GSH levels declined in response to knockdown of SNRPD2 or OTUD3 (Figure S12E,F). Moreover, cisplatin in combination with the knockdown of either gene more markedly suppressed colony formation (Figure S12G,H). These results support a model whereby reduced OTUD3 destabilizes SLC7A11, lowers antioxidant capacity, and thereby amplifies cisplatin‐induced oxidative stress, thus establishing a mechanistic basis for the observed chemosensitization. Hence, we determined whether iRGD‐Exo‐siS241F‐3/siSNRPD2 suppresses ovarian tumor growth and enhances the sensitivity to cisplatin. First, we evaluated whether the knockdown of both mtp53‐S241F and SNRPD2 reduces the half‐maximal inhibitory concentration (IC50) of cisplatin in ES‐2 cells. Our results showed that the depletion of both molecules led to a dramatic decrease in the IC50 of cisplatin from 42.61 to 12.90 µM (Figure 6G). Consistently, the colony formation assay also revealed that the depletion of both mtp53‐S241F and SNRPD2 significantly enhanced cisplatin sensitivity in ES‐2 cells (Figure 6H). Furthermore, we evaluated the anticancer effect of the engineered exosomes in vivo. ES‐2 cells were subcutaneously inoculated into five‐week‐old female BALB/c nude mice. Tumor‐bearing nude mice were randomly divided into five groups for different treatments: saline, cisplatin, iRGD‐Exo‐siNC, iRGD‐Exo‐siS241F‐3/siSNRPD2, and cisplatin combined with iRGD‐Exo‐siS241F‐3/siSNRPD2. Phosphate‐buffered saline (PBS) or cisplatin (2 mg kg^−1^) was administered intraperitoneally (i.p.) three times a week, while control exosomes or therapeutic exosomes were intratumorally (i.t.) administered three times a week (Figure 6I). Our results showed that individual treatment with either cisplatin or therapeutic exosomes significantly inhibited the growth rate (Figure 6J), weight (Figure 6K), and size (Figure 6L) of ES‐2‐derived xenograft tumors. Remarkably, the combination of cisplatin with the therapeutic exosomes resulted in a more substantial suppression of ovarian tumor growth (Figure 6J–L). No significant change in body weight was observed between control and treated mice, suggesting the potential adverse effects were tolerable (Figure 6M). In addition, to assess the translational potential of systemic delivery of engineered exosomes, we generated subcutaneous OVCAR‐3 xenografts (Figure S13A). Tail vein injection of the engineered exosomes loaded with siR248Q‐2—either alone or in combination with cisplatin—resulted in significant inhibition of tumor growth rate (Figure S13B), weight (Figure S13C), and size (Figure S13D), and no overt systemic toxicity (Figure S13E). Altogether, these findings demonstrate that dual targeting of mtp53 and SNRPD2 via engineered exosomes suppresses the growth of ovarian tumors and enhances their sensitivity to chemotherapy.

### 2.8 SNRPD2 is Highly Expressed in Ovarian Cancer and Associated With Poor Prognosis

**FIGURE 7:**
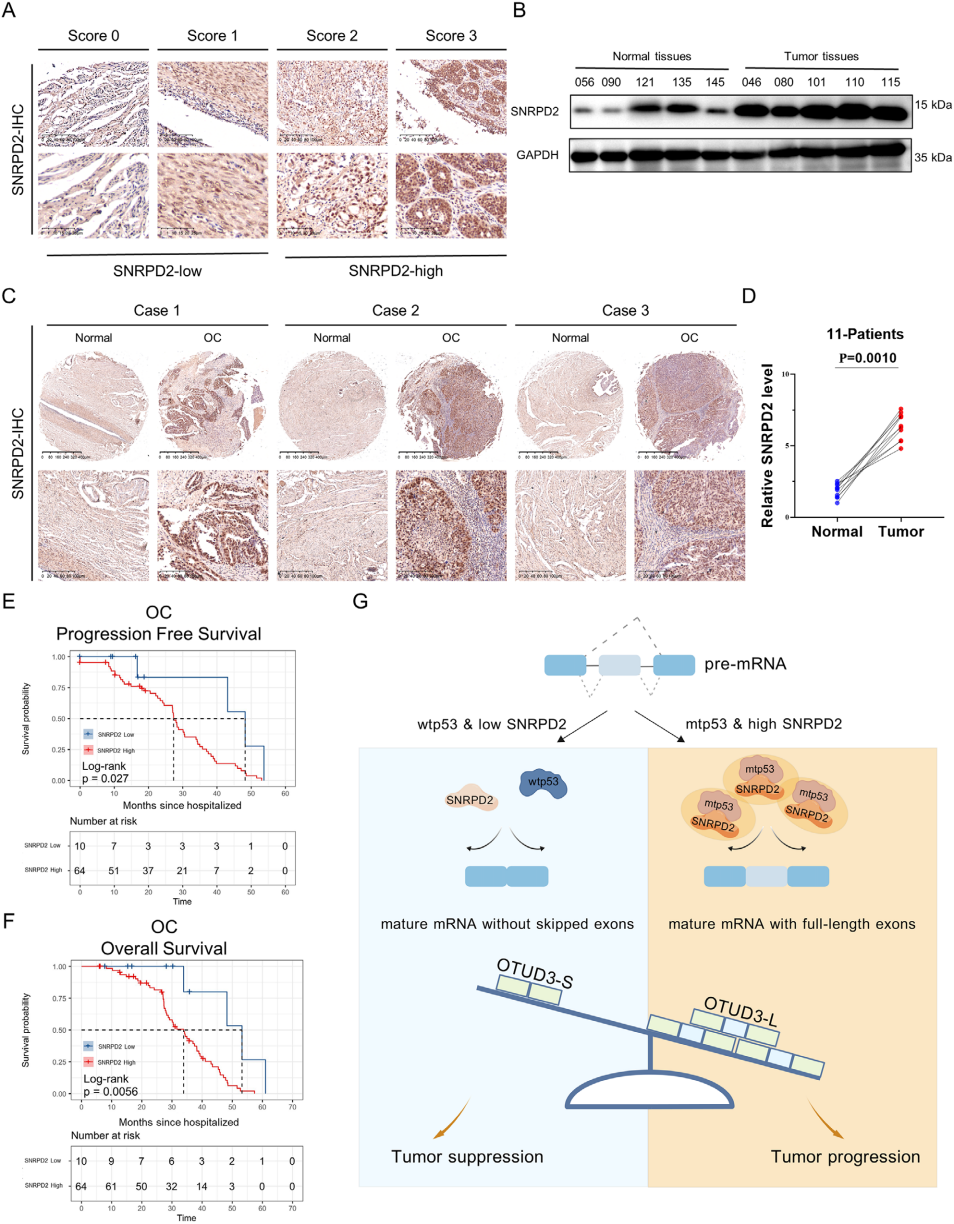
SNRPD2 is highly expressed in ovarian cancer and associated with poor prognosis. (A) Representative images of IHC staining of SNRPD2 in tissue microarray (TMA) of ovarian cancer patients, Scores 0 and 1 are categorized as negative; Scores 2 and 3 are considered positive. Scale bars: 100 µm (up), 25 µm (down). (B) IB analysis of SNRPD2 expression in normal fallopian tube samples and ovarian cancer samples. (C) Representative images of IHC staining of SNRPD2 in paired normal fallopian tube and ovarian cancer tissues. Scale bars: 400 µm (up), 100 µm (down). (D) Statistical analysis of SNRPD2 expression based on IHC scores in 11 pairs of matched tissues. (E,F) Higher levels of SNRPD2 are associated with worse prognosis in 74 patients with ovarian cancer. (G) A working model illustrates the role of SNRPD2 in concert with mtp53 during ovarian cancer progression.

Considering SNRPD2's oncogenic role and the fact that its targeted inhibition can impede ovarian cancer progression, we analyzed SNRPD2 expression in cancer and non‐cancerous tissues from TCGA and GTEx data to determine its clinical significance. The expression levels of SNRPD2 are significantly higher in a wide variety of cancers including ovarian cancer (Figure S14A,B). Additionally, the *SNRPD2* gene is frequently amplified in various cancers (Figure S14C). Furthermore, Kaplan‐Meier survival analysis showed that higher SNRPD2 levels correlated with poorer prognosis in patients with ovarian cancer (Figure S14D). In addition, we assessed SNRPD2 protein levels in normal fallopian tube and ovarian cancer specimens from Xiangya Hospital via immunohistochemistry (IHC) and IB analyses. Ovarian cancer samples showed a marked elevation of SNRPD2 levels compared with normal tissues (Figure 7A–D). Based on IHC scores, among 74 patients with HGSOC, those with high SNRPD2 expression exhibited a significantly advanced FIGO stage compared with the low‐expression group (Table S1). We further confirmed that higher SNRPD2 expression was significantly linked to worse progression‐free survival and overall survival of patients with ovarian cancer (Figure 7E,F). Therefore, these results emphasize the clinical significance of SNRPD2, a highly expressed oncogenic protein, further highlighting its potential as a therapeutic target in ovarian cancer.

## 3 Discussion

*TP53* mutations are highly prevalent in ovarian cancer, particularly in HGSOC, with up to 96% of HGSOC cases harboring *TP53* mutations that drive aggressive tumor behavior and therapeutic resistance [5, 6, 50, 51, 52]. In a clinically derived ovarian cancer sample harboring the p53‐S241F mutation, we identified SNRPD2 as a novel binding partner of mtp53 (Figure 1A). In addition to mtp53‐S241F, SNRPD2 also bound to other p53 mutants, such as R175H and R273H (Figure 1B–K). Interestingly, SNRPD2 failed to interact with wtp53 (Figure 1L,M), indicating that this interaction is pivotal for the GOF activity of mtp53s.

SNRPD2 is a core component of the spliceosomal small nuclear ribonucleoprotein (snRNP) complex, crucial for pre‐mRNA splicing [22, 24, 53]. It is essential for the assembly and stability of the U1, U2, U4, and U5 snRNPs, ensuring accurate spliceosome function and gene expression regulation, which is vital to rapidly proliferating cancer cells. In hepatocellular carcinoma (HCC), SNRPD2 is the most upregulated Sm protein and drives cancer progression [28]. Mechanistically, SNRPD2 cooperates with HNRNPL to regulate intron retention in DDX39A. This regulation leads to an increase in the expression of the short isoform (39A_S), which in turn mediates the nuclear export of MYC mRNA, thereby sustaining high MYC protein levels. Also, SNRPD2 modulates DNA damage in HCC through its impact on BRCA1/FANC cassette exons and expression, and the depletion of SNRPD2 sensitizes HCC cells to PARP inhibitors [54]. In this study, we found that mtp53 cooperates with SNRPD2 to facilitate the assembly of functional spliceosome (Figure 3; Figure S5), thereby promoting the levels of oncogenic transcripts that are critical for the growth and progression of ovarian cancer (Figure 4; Figures S8 and S9).

The interplay between SNRPD2 and mtp53 led to an increase in oncogenic transcripts and a concomitant decrease in tumor‐suppressive transcripts, profoundly orchestrating ovarian cancer progression. Specifically, the knockdown of mtp53 and SNRPD2 reduced OTUD3‐L expression and elevated OTUD3‐S expression (Figure 3E). OTUD3 belongs to the ovarian tumor (OTU) deubiquitinase (DUB) subfamily, which regulates various signaling pathways [39, 40]. Our results further revealed that OTUD3‐L promotes the proliferation, colony formation, and migration of ovarian cancer cells while attenuating apoptosis in these cells. In contrast, OTUD3‐S functions as a tumor suppressor to inhibit the progression of ovarian cancer (Figure 4A–H). These findings elucidate the mechanism by which mtp53 and SNRPD2 coordinately promote the progression of ovarian cancer through the regulation of alternative splicing. Intriguingly, it has been reported that OTUD3 may play either oncogenic or tumor‐suppressive roles in diverse human cancers [30, 55, 56, 57, 58]. Our study suggests that the levels of alternative splicing variants of OTUD3 may dictate its overall biological functions in either promoting or inhibiting tumorigenesis.

Targeting the spliceosome in tumors is a promising anticancer strategy, yet off‐target effects remain a critical challenge [21, 59]. SNRPD2 is significantly overexpressed in ovarian cancer and predicts poor clinical outcomes (Figure 7; Figure S14), suggesting its role as a druggable target for modulating spliceosome activity in precision therapy. Mtp53 functions as an oncogenic driver in ovarian carcinogenesis, thereby representing a compelling therapeutic target. We employed a screening strategy and identified a specific siRNA that can achieve allele‐specific silencing while preserving wtp53 expression in normal cells (Figure 5). Our results further demonstrated that the co‐depletion of SNRPD2 and mtp53 exerts a more potent tumor‐suppressive effect (Figure S9). More importantly, therapeutic exosomes containing siRNAs specific to mtp53 and SNRPD2 were developed. We used HEK293T cells as donors for exosome production primarily because of their well‐established advantages, including high exosome yield, excellent transfection efficiency for cargo loading, ease of genetic engineering for targeted modification, and consistent batch‐to‐batch reproducibility. These engineered exosomes were shown to effectively suppress the growth of ovarian cancer cells and enhance their sensitivity to chemotherapy in vivo (Figure 6I–M; Figure S13A–E). Our study presents a multi‐targeted therapeutic approach for cancer treatment, which may offer a promising strategy for advancing precision therapy in ovarian cancer.

In conclusion, our study uncovers SNRPD2 as a binding partner of mtp53. SNRPD2 is highly expressed in ovarian cancer and associated with a poor prognosis. Consistently, the overexpression of SNRPD2 promotes whereas the knockdown of SNRPD2 inhibits the growth and migration of ovarian cancer cells. Mechanistic study reveals that mtp53 cooperates with SNRPD2 to facilitate the assembly of Sm/SMN protein complex. Moreover, co‐targeting mtp53 and SNRPD2 diminishes the levels of oncogenic transcripts (e.g., OTUD3‐L) while elevating those of their tumor‐suppressive counterparts (e.g., OTUD3‐S). Finally, therapeutic engineered exosomes encapsulating siRNAs selectively targeting mtp53 and SNRPD2 demonstrate significant tumor suppression in ovarian cancer, with specificity to mtp53 and no off‐target effects on wild‐type p53.

## 4 Materials and Methods

### 4.1 Plasmids and Antibodies

The Flag‐tagged SNRPD2‐expressing plasmid was purchased from Vigene Biosciences, Inc. The Myc‐tagged SNRPD2 plasmid was generated by inserting the full‐length cDNA amplified by PCR into the pcDNA3.1/Myc‐His vector between the EcoRI and BamHI sites, using the designed primers (Table S2). The plasmids encoding non‐tagged mtp53s (R175H, S241F, and R273H) were generated as previously described [13]. The detailed sequences of shRNA targeting SNRPD2 were obtained from Sigma–Aldrich (Table S3). The following antibodies were commercially obtained: the anti‐Myc (Cat. No. 60003‐1‐Ig, Proteintech), anti‐FLAG (Cat. No. F1804, Sigma–Aldrich, St. Louis, MO, USA), anti‐p53 (Cat. No. sc‐126, DO‐1, Santa Cruz Biotechnology), anti‐SNRPD2 (Cat. No. 14789‐1‐AP, Proteintech), anti‐Tubulin (Cat. No. 66031‐1‐Ig, Proteintech), and anti‐GAPDH (Cat. No. 60004‐1‐Ig, Proteintech). Secondary antibodies included HRP‐conjugated goat anti‐rabbit IgG (Cat. No. ARG65351, Arigo), HRP‐conjugated goat anti‐mouse IgG (Cat. No. ARG65350, Arigo), and HRP‐conjugated goat anti‐mouse light chain‐specific antibody (Cat. No. 115‐035‐174, Jackson ImmunoResearch). Proteins were visualized using the ECL chemiluminescence reagent (Yeasen, Shanghai, China).

### 4.2 Cell Culture and Transient Transfection

Human ovarian cancer cell lines, ES‐2^p53‐S241F^, OVCA420^p53‐R273H^, TOV112D ^p53‐R175H^, SKOV3^p53‐R273H^ [14], SKOV3^p53‐null^, A2780^wtp53^, and HEY ^wtp53^, colon cancer cell line HCT116^p53‒/‒^, and HEK293T were cultured in Dulbecco's modified Eagle's medium (DMEM) supplemented with 10% FBS (Yeasen, Shanghai, China), 100 U mL^−1^ penicillin, and 100 µg mL^−1^ streptomycin ((BasalMedia, Shanghai, China). OVCAR‐3^p53‐R273H^ cells were maintained in RPMI 1640 medium supplemented with 10% FBS (Yeasen, Shanghai, China), 100 U mL^−1^ penicillin, 100 µg mL^−1^ streptomycin ((BasalMedia, Shanghai, China), and an additional 0.01 mg mL^−1^ human recombinant insulin (Yeasen, Shanghai, China). All cells were cultured at 37°C in an incubator containing 5% CO2. Cells were seeded in the plates at an appropriate density one day before transfection according to the manufacturer's protocol of the Hieff Trans liposomal transfection reagent (Yeasen) [60, 61]. Specifically, plasmid DNA and transfection reagent were diluted separately in serum‐free medium and sequentially incubated at room temperature as follows. For transfection in 10 cm dishes, 10 µg plasmid DNA (1:1 mass ratio for two‐plasmid co‐transfection) was diluted in 1 mL medium, and 25 µL Hieff Trans reagent was diluted in a second 1 mL aliquot; For 6‐well plates, 2–3 µg plasmid DNA or 5–10 µL of 20 µM siRNA was diluted in 250 µL medium, and 5–10 µL Hieff Trans reagent was diluted in a separate 250 µL aliquot. Each dilution was incubated for 5 min, the two aliquots were combined, gently mixed, and incubated for an additional 20 min. The complexes were added directly to the cultures; dishes were gently rocked to ensure homogeneous distribution, and cells were maintained at 37°C under 5% CO_2_. If pronounced cytotoxicity was observed, the medium was replaced 4–6 h post‐transfection. Cells were then split or collected for further experiments 24–96 h post‐transfection. The detailed sequences of siRNAs were provided in Table S4.

### 4.3 Immunoblotting (IB)

Cells were lysed using lysis buffer containing 50 mM Tris/HCl (pH 7.5), 0.5% Nonidet P‐40 (NP‐40), 1 mM ethylenediaminetetraacetic acid (EDTA), 15  mM NaCl, 1 mM dithiothreitol (DTT), 0.2 mM phenylmethylsulfonyl fluoride (PMSF), 10 mM pepstatin A, 1 mM leupeptin, and 10% protease inhibitor cocktail. Equal amounts of clear cell lysate were used for IB analysis as described previously [13]. Protein expression was detected using primary antibodies against the anti‐Myc (Cat. No. 60003‐1‐Ig, Proteintech), anti‐FLAG (Cat. No. F1804, Sigma‐Aldrich, St. Louis, MO, USA), anti‐p53 (Cat. No. sc‐126, DO‐1, Santa Cruz Biotechnology), anti‐SNRPD2 (Cat. No. 14789‐1‐AP, Proteintech) at a dilution of 1:1000, anti‐Tubulin (Cat. No. 66031‐1‐Ig, Proteintech), and anti‐GAPDH (Cat. No. 60004‐1‐Ig, Proteintech) at a dilution of 1:5000.

### 4.4 Immunoprecipitation

The immunoprecipitation (IP) assay [13] was performed using antibodies as indicated in the figure legends. Briefly, 500–1000 µg of protein lysate was incubated with the indicated antibody (1 µg antibody per 500 µg protein lysate) at 4°C for 4–10 h. Protein A or G beads (Santa Cruz Biotechnology) were then added, and the mixture was incubated at 4 °C for additional 1–3 h. The beads were washed 3–6 times with lysis buffer. Bound proteins and 10% input samples were detected by IB using antibodies as indicated in the figure legends. The proteasome inhibitor MG132 (Sigma–Aldrich) was added 4–6 h before cells were harvested for the IP assay.

### 4.5 Generation of Stable Cell Lines

For stable overexpression or knockdown, lentiviral particles were produced in HEK293T cells co‐transfected with pCDH‐Flag‐SNRPD2 (or pLKO.1‐tet‐on‐shSNRPD2), psPAX2 and pMD2.G at a mass ratio of 5:3:2. Supernatants were collected at 48 h, filtered (0.45 µm) and used to infect ES‐2 or TOV112D cells in the presence of 8 µg mL^−1^ polybrene. Pools were selected with 1 µg mL^−1^ puromycin for 10 days [14]. Stable lines are referred to as PCDH‐SNRPD2 or tet‐shSNRPD2 throughout the manuscript.

### 4.6 CRISPR/Cas9‐Mediated Gene Editing

For generation of the p53‐null cell line, the CRISPR/Cas9 system (lentiCRISPRv2 vector, Addgene) was used. High‐scoring sgRNAs targeting p53 were designed as previously mentioned [13, 62], and the annealed sgRNA oligos (compatible with BsmBI digestion) were cloned into the lentiCRISPRv2 vector. Lentiviral particles were produced as described above, then used to infect target cells (ES‐2) in the presence of 8 µg mL^−1^ polybrene. Infected cells were selected with 1 µg mL^−1^ puromycin for >7 days to obtain stable polyclonal populations.

### 4.7 Cell Viability Assay

The Cell Counting Kit‐8 (CCK‐8; Yeasen) was used according to the manufacturer's protocol. Cell suspensions were seeded at a density of 2500–5000 cells per well in 96‐well culture plates 12–24 h post‐transfection. CCK‐8 reagent was added to the cultures at a final concentration of 10% and incubated for 2–4 h. The absorbance of the samples at 450 nm was measured daily for 5 days using a microplate reader [13].

### 4.8 Colony Formation Assay

500–1500 cells were seeded into 6‐well plates (triplicate wells per group) and cultured in complete growth medium at 37°C with 5% CO_2_ for 7–14 days, with medium refreshed every 3–4 days to maintain optimal nutrient conditions. Following incubation, colonies were washed with phosphate‐buffered saline (PBS), fixed with 4% paraformaldehyde for 15–30 m, and stained with 0.5% crystal violet for 10–30 m. The stained colonies were washed gently with distilled water, air‐dried, and counted manually or quantified using ImageJ software [13].

### 4.9 Flow Cytometric Analysis of Apoptosis

Apoptosis was assessed using the Annexin V‐PE/7‐AAD Apoptosis Detection Kit (Vazyme) in strict accordance with the manufacturer's instructions with appropriate operational optimizations for cell type compatibility [14, 48]. Briefly, 2–5 × 10^5^ cells were harvested and centrifuged at 300 × g for 5 min at 4°C, followed by two washes with pre‐cooled phosphate‐buffered saline (PBS) to remove residual culture medium and debris. After the final wash, cell pellets then resuspended in 1× Binding Buffer (provided in the kit) at a density of 1 × 10^6^ cells mL^−1^. Next, 5 µL of Annexin V‐PE and 5 µL of 7‐aminoactinomycin D (7‐AAD) were added to the cell suspension and mixed thoroughly, then incubated at room temperature for 15 min in complete darkness to prevent fluorophore quenching and ensure specific probe binding. Post‐incubation, the samples were immediately analyzed using a CytoFLEX S flow cytometer (Beckman Coulter, Indianapolis, IN, USA). Data acquisition was performed with CytExpert software (Beckman Coulter) and apoptotic cells were defined as Annexin V‐PE^+^/7‐AAD^−^ (early apoptosis) or Annexin V‐PE^+^/7‐AAD^+^ (late apoptosis); the total percentage of apoptotic cells was calculated for statistical comparison.

### 4.10 Cell Migration Assay

5–10 × 10^4^ cells suspended in 100 µL of serum‐free medium were added to upper chambers, while lower chambers were filled with normal culture medium. After incubation at 37°C for 24–48 h, cells on the upper surface were scraped off and washed away, cells on the lower surface were fixed with methanol and stained with 0.2% crystal violet for 2–6 h. The number of migratory cells was counted in at least three randomly selected fields under an optical microscope using ImageJ software [48].

### 4.11 Wound‐Healing Assay

5–10 × 10^5^ cells were cultured in 6‐well plates until they reached a confluence of 90%–100%. Using a sterile 200 µL pipette tip, a straight scratch was made across the center of each well to create a “wound” area. The floating cells and debris generated during the scratching process were carefully removed by washing the wells three times with pre‐warmed PBS. Then, 2 mL of serum‐free medium was added to each well. The plates were incubated at 37°C in a 5% CO_2_ incubator. Images of the “wound” area in each well were captured immediately after scratching (time 0 h) and at specific time intervals (e.g., 12, 24, and 36 h) using an inverted microscope with a 10 × objective lens. To quantify the wound closure rate, the width of the “wound” area in each captured image was measured using ImageJ software. The percentage of wound closure was calculated according to the formula: [(initial wound width—wound width at a specific time point) / initial wound width] × 100%. The experiment was performed in triplicate (*n* = 3) for each condition, and the average values were used for statistical analysis [13].

### 4.12 Glutathione Assay

Glutathione (GSH) content was determined with the commercial GSH assay kit (Nanjing Jiancheng Bioengineering Institute) according to the manufacturer's instructions. In brief, 48 h after siRNA transfection, the cells were harvested by trypsinization, washed twice with ice‐cold PBS, and lysed by sonication on ice for 2 min. The lysate was centrifuged at 3500 rpm for 10 min, and the supernatant was immediately mixed with the kit reagents. Absorbance was read at 420 nm with a microplate reader [63].

### 4.13 Reverse Transcription, Quantitative PCR and PCR

Total RNA was extracted from ES‐2, OVCA420, and TOV112D cells using RNAiso Plus and quantified with a NanoDrop spectrophotometer. Reverse transcription was carried out using HiScript III RT SuperMix (Vazyme, Nanjing, China) according to the manufacturer's instructions [13, 62]. The resultant cDNA was stored at −20°C for the subsequent use. Quantitative PCR (qPCR) was performed with SYBR Green qPCR Master Mix (Vazyme) following the manufacturer's instructions. Relative mRNA abundance was calculated by the 2^−ΔΔCt^ method and normalized to GAPDH [62]. PCR was performed using 2 × Phanta UniFi Master Mix (Vazyme) [64] to amplify the target gene following the manufacturer's protocol. PCR products were analyzed by electrophoresis on a 2% agarose gel stained with YeaRed Nucleic Acid Gel Stain (Yeasen) and visualized under UV light. The sequences of the primers used in this study were listed in Table S5.

### 4.14 RNA‐Sequencing

Ovarian cancer cells transfected with siNC, siSNRPD2, or sip53 for 48 h were collected. Total RNA was isolated using RNAiso Plus according to the manufacturer's protocol (Takara, Japan). RNA‐sequencing was provided by OEbiotech (Shanghai, China) [48]. Complete gene‐level statistics (log2FC, *p* values, FDR, counts) were provided in Table S6.

### 4.15 Immunofluorescence Staining

5–10 × 10^4^ cells were incubated with Exo‐PKH67 or iRGD‐Exo‐PKH67 for 8 h, followed by fixation with methanol at −20°C overnight. The cells were subsequently washed three times with PBS. After washing, nuclear staining was performed by incubating the cells with 4’,6‐diamidino‐2‐phenylindole (DAPI, Sigma–Aldrich). Fluorescence images were captured using an inverted fluorescence microscope (Leica, Wetzlar, Germany) [48].

### 4.16 Immunohistochemistry

Tissue samples were collected and fixed in 10% neutral buffered formalin, then embedded in paraffin. Tissue microarray sections were mounted on glass slides, deparaffinized in xylene, and rehydrated through a graded ethanol series (100%, 95%, 80%, and 70%) to distilled water. Antigen retrieval was performed using Sodium citrate‐EDTA antigen repair solution (cat. No. P0086, Beyotime) according to the manufacturer's instructions, followed by cooling at room temperature for 60 min. After washing in PBS for 30 min at room temperature, tissues were incubated with a primary antibody for 1 h at room temperature. Following extensive PBS washing, tissues were incubated with a secondary antibody (cat. No. GK500705, GeneTech) for 1 h at room temperature. The staining signal was visualized using 3,3’‐diaminobenzidine (DAB; cat. No. GK500705, GeneTech) for 5 m, followed by hematoxylin counterstaining. Staining density was measured under a microscope and quantified using ImageJ [48].

### 4.17 Exosome Isolation, Purification and Loading With siRNA

Exosomes were harvested from the culture medium of HEK293T cells transfected with plasmids encoding the iRGD‐LAMP2B fusion protein, as previously described [48]. The medium was first centrifuged at 300 × g for 10 m. The supernatant was collected and centrifuged at 10 000 × g for 30 m to remove cells and large debris, after which the supernatant was transferred to ultracentrifuge tubes. Exosomes and contaminating proteins were pelleted by ultracentrifugation at 100 000 × g for 70 m. The pellet was washed with PBS and ultracentrifuged again at 100 000 × g centrifugation for 70 m. Transmission electron microscopy (TEM) and nanoparticle tracking analysis (NTA) services were provided by Umibio Co. Ltd (Shanghai).

The siRNA with 2’‐O‐methyl modification was purchased from GenePharma (Shanghai, China). For siRNA encapsulation, the electroporation cup was rinsed at least three times with ultrapure water, followed by three rinses with 75% ethanol, and then air‐dried in a laminar flow hood. Then, siRNA was added to the exosomes at a ratio of 1:2 and gently mixed to form the Exo‐siRNA mixture. Electroporation was performed with parameters set as follows: 400 V, 200 Ω, 125 µF, and a single pulse was delivered. The mixture was transferred into the dried electroporation cup, which was then placed into the electroporation chamber. The encapsulation efficiency of siRNA in exosomes was approximately 5%. Immediately after electroporation, the cup was ice‐bathed for 5 m. The mixture was filtered through a 0.22 µm filter, aliquoted at 0.5 mg per tube and store at −80°C for future use.

### 4.18 Mouse Xenograft Experiments

Four‐ to six‐week‐old female BALB/c nude mice were obtained from Laboratory Animal Science, Fudan University Shanghai Cancer Center. To assess the in vivo effect of SNRPD2 on tumor growth, mice were subcutaneously inoculated in each flank with 5 × 10^6^ ES‐2 cells stably expressing PCDH vector, PCDH‐SNRPD2, tet‐shNC, or tet‐shSNRPD2 per flank [13, 48]. The stably transfected cells for mouse xenograft assays were generated according to protocols described above. Once tumors in the tet‐shNC and tet‐shSNRPD2 groups were visible, doxycycline (DOX) was added to the drinking water as indicated. Tumor growth was monitored every other day in two dimensions using electronic digital calipers. Tumor volume was calculated using the formula: volume  =  length × width^2^ × 0.5. Tumors were then harvested, weighed, and measured.

To investigate the antitumor effect of iRGD‐Exo‐siS241F‐3/siSNRPD2, mice were subcutaneously inoculated in each flank with 7 × 10^6^ ES‐2 cells, and exosomes (100 µg per injection) were intratumorally injected into tumor‐bearing mice three times a week. To explore the combined therapeutic efficacy of iRGD‐Exo‐siS241F‐3/siSNRPD2 with chemotherapy, tumor‐bearing mice were randomly divided into five groups for different treatments: saline; iRGD‐Exo‐siNC; iRGD‐Exo‐siS241F‐3/siSNRPD2; 2 mg kg^−1^ cisplatin (DDP); and 2 mg kg^−1^ DDP combined with iRGD‐Exo‐siS241F‐3/siSNRPD2. Each 100 µg dose of exosomes contained 2.5 µg siNC (iRGD‐Exo‐siNC) or 2.5 µg siS241F‐3 and siSNRPD2 (iRGD‐Exo‐siS241F‐3/siSNRPD2). Saline and DDP were intraperitoneally administered, while engineered exosomes were delivered intratumorally. To investigate the antitumor effect of iRGD‐Exo‐siR248Q‐2/siSNRPD2 with chemotherapy, mice were subcutaneously inoculated with 1 × 10^7^ OVCAR‐3 cells in each flank, and exosomes (100 µg per injection) were intravenously injected via the tail vein into tumor‐bearing mice three times a week. Tumor‐bearing mice were randomly divided into five groups for different treatments: saline; iRGD‐Exo‐siNC; iRGD‐Exo‐siR248Q‐2/siSNRPD2; 3 mg kg^−1^ cisplatin (DDP); and 3 mg/kg DDP combined with iRGD‐Exo‐siR248Q‐2/siSNRPD2. Saline and DDP were intraperitoneally administered, while engineered exosomes were delivered intravenously. When tumors reached an appropriate volume, the nude mice were sacrificed, and tumors were harvested, weighed, and measured. These studies were approved by the Animal Welfare Committee of Fudan University Shanghai Cancer Center (FUSCC‐IACUC‐2024327, FUSCC‐IACUC‐2025007, and FUSCC‐IACUC‐2025610).

### 4.19 Database Analysis of Cancer Patients

The expression of SNRPD2 in cancer versus relative normal samples was analyzed using Sangerbox tools [65] (http://www.sangerbox.com/tool). Cancer patients’ prognosis was evaluated by Kaplan‐Meier survival analysis [66] (kmplot.com). Specimens in this study were collected from ovarian cancer patients who underwent laparoscopy or primary debulking surgeries at Xiangya Hospital of Central South University in China between 2018 and 2023. All samples were approved by the Medical Ethics Committee of Xiangya Hospital, Central South University (202208204‐2). Written informed consent was obtained from all participants. Statistical significance was set at *p* < 0.05. Significance levels are denoted by asterisks: * *p* < 0.05, ** *p* < 0.01. Quantitative data were presented as mean ± standard deviation (SD).

### 4.20 Statistical Analysis

All in vitro experiments were conducted in biological triplicate. Animal cohorts for in vivo experiments were randomly allocated to different groups. All data are expressed as mean ± standard deviation (SD). Statistical analyses were performed using GraphPad Prism 8.0. After confirming normality, data were analyzed with two‐tailed unpaired Student's *t*‐test (comparison of two groups) or one‐way ANOVA followed by Dunnett's multiple‐comparisons test (comparison of more than two groups). Kaplan–Meier method and log‐rank test were used to assess differences in patient survival. Clinicopathologic associations were evaluated using χ^2^ test or Fisher's exact test, where appropriate. A *p*‐value < 0.05 was considered statistically significant. Asterisks denote statistical significance: *p < 0.05; **p < 0.01.

## Author Contributions

**W.Z**. and **Q.H**. contributed equally to this work. **W.Z**. and **Q.H**. conducted the experiments and analyzed the data; **Y.G**. and **J.T**. conducted part of the experiments; **X.C**. and **S.T**. assisted with tissue microarray analysis; **R.R**., **Y.H**., **M.C**., and **J.D**. analyzed part of the data; **T.H**., **G.S**., and **B.G**. assisted with the isolation and purification of exosomes; **Q.H**., **B.G**., **Y.Z**., and **X.Z**. conceived, designed and supervised the study and analyzed the data; **W.Z**., **Y.Z**., and **X.Z**. wrote the paper.

## Conflicts of Interest

The authors declare no conflicts of interest.

## Supporting information




**Supporting File**: advs73835‐sup‐0001‐SuppMat.pdf.

**Supporting File**: advs73835‐sup‐0002‐TableS6.xlsx.

## Data Availability

The data that support the findings of this study are available from the corresponding author upon reasonable request.
