# Correction to “Dual Targeting of Mutant p53 and SNRPD2 via Engineered Exosomes Modulates Alternative Splicing to Suppress Ovarian Cancer”

**DOI:** 10.1002/advs.75571

**Published:** 2026-05-07

Zhao W, Hao Q, Gan Y, Tong J, Chen X, Tan S, Ruan R, Huang Y, Cao M, Deng J, Han T, Shi G, Gao B, Zhang Y, Zhou X, “Dual Targeting of Mutant p53 and SNRPD2 via Engineered Exosomes Modulates Alternative Splicing to Suppress Ovarian Cancer,” *Adv* *anced* *Sci* *ence* (Weinh). 2026 Mar;13(17):e13369. https://doi.org/10.1002/advs.202513369. Epub 2026 Jan 20. PMID: 41560375; PMCID: PMC13042878.

Wei Zhao^1,2,3,#^, Qian Hao^4,5,#^, Yu Gan^4,5^, Jing Tong^4,5^, Xiaodan Chen^1,2,3^, Shuran Tan^1,2,3^, Ruiwen Ruan^6,7^, Yingdan Huang^4,5^, Mingming Cao^4,5^, Jun Deng^6,7^, Tao Han^8^, Getao Shi^9^, Bo Gao^9,*^, Yu Zhang^1,2,3,*^, and Xiang Zhou^4,5,10,*^

^1^Department of Gynecology, Xiangya Hospital, Central South University, Changsha, China

^2^Gynecological Oncology Research and Engineering Center of Hunan Province, Changsha, China

^3^National Clinical Research Center for Geriatric Disorders, Xiangya Hospital, Central South University, Changsha, China

^4^Fudan University Shanghai Cancer Center and Institutes of Biomedical Sciences, Fudan University, Shanghai, China

^5^Department of Oncology, Shanghai Medical College, Fudan University, Shanghai, China

^6^Department of Oncology, The First Affiliated Hospital, Jiangxi Medical College, Nanchang University, Nanchang, Jiangxi, China

^7^Jiangxi Key Laboratory for Individual Cancer Therapy, Nanchang, Jiangxi, China

^8^Xinxiang Key Laboratory for Molecular Oncology, Institutes of Health Central Plains, Xinxiang Medical University, Xinxiang, China

^9^Umibio Co. Ltd., Shanghai, China

^10^Key Laboratory of Breast Cancer in Shanghai, Department of Breast Surgery, Fudan University Shanghai Cancer Center, Fudan University, Shanghai, China

^#^ Equal contribution

^*^Correspondence:

Xiang Zhou, Email: xiangzhou@fudan.edu.cn

Yu Zhang, Email: xyzhangyu@csu.edu.cn

Bo Gao, Email: bogao@umibio.cn

In Figure 2P of the original published manuscript, the flow cytometry panel for SKOV3 ^R273H^ was erroneously duplicated from Figure 2M during the figure compilation process, and the authentic experimental flow cytometry panel was accidentally excluded from the final published version.

The corrected full high‐resolution Figure 2 is shown below:


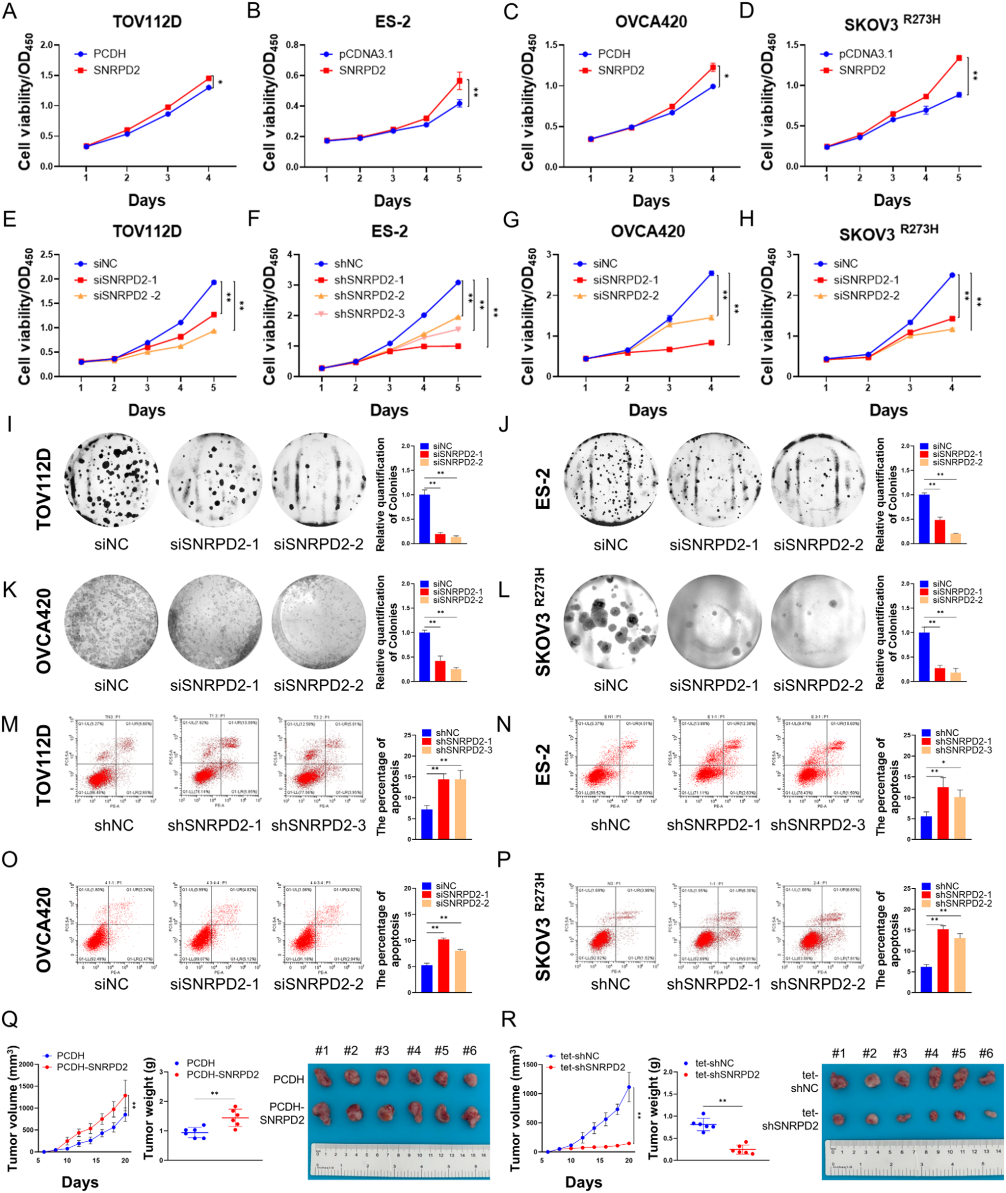


This graphical error arose from an unintentional oversight during figure arrangement. It does not alter any quantitative experimental data, statistical analyses, core research findings, or the overall scientific conclusions of the study. All authors have thoroughly examined and formally endorsed this correction.

All other content and results of the original article remain completely unchanged, and the integrity of the study conclusions remains unaffected.

We apologize for this error.

