# Supplementary material for: Dual Targeting of Mutant p53 and SNRPD2 via Engineered Exosomes Modulates Alternative Splicing to Suppress Ovarian Cancer
Source: Adv Sci (Weinh). 2026 Jan 20;13(17):e13369. doi: 10.1002/advs.202513369 (PMC13042878; doi:10.1002/advs.202513369)
Supplement: Supplementary file 1 — Supporting File: advs73835‐sup‐0001‐SuppMat.pdf. [file ADVS-13-e13369-s001.pdf]

Supplementary Information

Dual targeting of mutant p53 and SNRPD2 via engineered exosomes modulates alternative splicing to suppress ovarian cancer

Wei Zhao[1,2,3,#], Qian Hao[4,5,#], Yu Gan[4,5], Jing Tong[4,5], Xiaodan Chen[1,2,3], Shuran Tan[1,2,3], Ruiwen Ruan[6,7], Yingdan Huang[4,5], Mingming Cao[4,5], Jun Deng[6,7], Tao Han[8], Getao Shi[9], Bo Gao[9,*], Yu Zhang[1,2,3,*], and Xiang Zhou[4,5,10,*]

[1] Department of Gynecology, Xiangya Hospital, Central South University, Changsha, China
[2] Gynecological Oncology Research and Engineering Center of Hunan Province, Changsha, China
[3] National Clinical Research Center for Geriatric Disorders, Xiangya Hospital, Central South University, Changsha, China
[4] Fudan University Shanghai Cancer Center and Institutes of Biomedical Sciences, Fudan University, Shanghai 200032, China
[5] Department of Oncology, Shanghai Medical College, Fudan University, Shanghai 200032, China
[6] Department of Oncology, The First Affiliated Hospital, Jiangxi Medical College, Nanchang University, Nanchang 330006, Jiangxi, China
[7] Jiangxi Key Laboratory for Individual Cancer Therapy, Nanchang 330006, Jiangxi, China
[8] Xinxiang Key laboratory for Molecular Oncology, Institutes of Health Central Plains, Xinxiang Medical University, Xinxiang 453003, China
[9] Umibio Co. Ltd., Shanghai 201210, China
[10] Key Laboratory of Breast Cancer in Shanghai, Department of Breast Surgery, Fudan University Shanghai Cancer Center, Fudan University, Shanghai, 200032, China

[#] Equal contribution
[*] Correspondence:
Xiang Zhou, Email: xiangzhou@fudan.edu.cn
Yu Zhang, Email: xyzhangyu@csu.edu.cn
Bo Gao, Email: bogao@umibio.cn

Running title: Co-targeting mutant p53 and SNRPD2 in ovarian cancer

Supplementary figure legends

Figure S1. SNRPD2 is a mutant p53-binding protein in ovarian cancer. Related to Figure 1.

(A-F) HCT116 $^{p53-/-}$ cells were transfected with the indicated plasmids, followed by co-IP-IB analysis using antibodies as indicated.

(G-I) TOV112D (mtp53-R175H), ES-2 (mtp53-S241F), and OVCA420 (mtp53-R273H) cells were treated with MG132 (20 μM) for 6 h, followed by co-IP-IB analysis.

(J) HCT116 $^{p53-/-}$ cells were transfected with the indicated plasmids, followed by co-IP-IB analysis.

(K) HEYA8 (wtp53) cells were treated with MG132 (20 μM) for 6 h, followed by co-IP-IB analysis.

Figure S2. SNRPD2 promotes ovarian cancer proliferation in vitro. Related to Figure 2.

(A and B) ES-2 (A) and OVCA420 (B) cells were transfected with the indicated plasmids, followed by the colony formation assay.

Data are represented as mean ± SD, $n = 3$. ** $p < 0.01$.

Figure S3. SNRPD2 promotes ovarian cancer migration in vitro. Related to Figure 2.

(A-H) TOV112D, ES-2, OVCA420, and SKOV3 $^{p53\text{-}R273H}$ cells were transfected with the plasmids, siRNAs, or shRNAs as indicated, followed by the wound healing assay.

(I-N) TOV112D, ES-2, and SKOV3 $^{p53\text{-}R273H}$ cells were transfected with the plasmids or shRNAs as indicated, followed by the cell migration assay.

Data are represented as mean ± SD, $n = 3$. ** $p < 0.01$.

Figure S4. Schematic of siRNA transfection and RNA sequencing workflow in OVCA420 cells. Related to Figure 3.

(A) OVCA420 cells were transfected with the indicated siRNAs, followed by RNA sequencing. A Venn diagram shows the overlap of different splicing genes (DSG) between the sip53 versus siNC and siSNRPD2 versus siNC groups.

(B) OVCA420, TOV112D, and ES-2 cells were transfected with the indicated siRNAs, followed by RT-PCR analysis.

Figure S5. Mutant p53 collaborates with SNRPD2 to modulate splicing switch. Related to Figure 3.

(A) Gene Ontology analysis of genes undergoing SE events upon the depletion of p53 or SNRPD2 in OVCA420 cells.

(B) HCT116 $^{p53-/-}$ cells were transfected with the indicated plasmids, followed by co-IP-IB analysis using antibodies as indicated.

(C) Schematic illustration of the interaction between SMN and SNRPD2 in the presence or absence of mtp53 involvement.

Figure S6. Mutant p53 collaborates with SNRPD2 to modulate splicing switch. Related to Figure 3.

(A) ES-2 or ES-2 $^{p53-/-}$ (generated by CRISPR/Cas9) cells were transfected with the indicated plasmids, followed by co-IP-IB analysis.

(B) ES-2 or ES-2 $^{p53-/-}$ cells were transfected with the indicated plasmids, followed by co-IP-IB analysis.

(C-F) OVCA420, TOV112D, and ES-2 cells were transfected with the indicated siRNAs, followed by RT-PCR analysis. ImageJ software was used to quantify the band intensity.

Figure S7. The impact of OTUD3, EAF2, GSTO2, or FOCAD knockdown on proliferation in ovarian cancer cells. Related to Figure 3.

(A-D) TOV112D and ES-2 cells were transfected with indicated siRNAs, followed by RT-qPCR analysis and the cell viability assay (A-D).

Data are represented as mean ± SD, $n = 3$. * $p < 0.05$, ** $p < 0.01$, ns, not significant.

Figure S8. Overexpression of both mutant p53 and SNRPD2 cooperatively promotes ovarian cancer progression. Related to Figure 3.

(A-F) Cells were transfected with control, SNRPD2, mtp53-R273H, or combined SNRPD2 plasmid and mtp53-R273H plasmid as indicated for 24 h, followed by the cell viability assay (A and B), colony formation assay (C and D), and cell migration assay (E and F).

(G-H) Cells were transfected with control, SNRPD2, mtp53-R273H, or combined SNRPD2 plasmid and mtp53-R273H plasmid as indicated for 48–96 h, followed by flow cytometric analysis (G and H).

Data are represented as mean ± SD, $n = 3$. * $p < 0.05$, ** $p < 0.01$. Scale bars in E and F: 100 μm.

Figure S9. Ablation of both mutant p53 and SNRPD2 synergistically suppresses ovarian cancer. Related to Figure 3.

(A-F) Cells were transfected with control, SNRPD2 siRNA, p53 siRNA, or combined SNRPD2 siRNA and p53 siRNA as indicated for 24 h, followed by the cell viability assay (A and B), colony formation assay (C and D), and cell migration assay (E and F)

(G-H) Cells were transfected with control, SNRPD2 siRNA, p53 siRNA, or combined SNRPD2 siRNA and p53 siRNA as indicated for 48–96 h, followed by flow cytometric analysis (G and H).

Data are represented as mean ± SD, $n = 3$. * $p < 0.05$, ** $p < 0.01$. Scale bars in E and F: 100 μm.

Figure S10. Design of siRNAs specific to mutant p53-R248Q without wild-type p53 interference. Related to Figure 5.

(A) siRNA sequences specifically targeting mtp53- R248Q. The mutated nucleotide residue is highlighted.

(B-E) OVCAR-3 (B), OVCA420 (C), A2780 (D), and HEY (E) cells were transfected with indicated siRNAs, followed by IB analysis.

(F and G) OVCAR-3 (F) and HEY (G) cells were transfected with indicated siRNAs, followed by the cell viability assay.

Data are represented as mean ± SD, $n = 3$. ** $p < 0.01$, ns, not significant.

Figure S11. Characterization of engineered exosomes. Related to Figure 6.

(A) A representative TEM image of exosomes. Scale bar: 200 nm.

(B) Particle size distribution of exosomes.

(C) 293T cells and exosomes were collected for IB analysis using antibodies as indicated. The exosomal markers TSG101 and CD63 were detected, the endoplasmic reticulum calnexin was used as a reference for comparison.

Figure S12. SNRPD2/OTUD3 ablation reduces SLC7A11/GSH and sensitizes ovarian cancer cells to cisplatin. Related to Figure 6.

(A-D) ES-2 and TOV112D cells were transfected with siRNAs as indicated for 48 h, followed by RT-qPCR and IB analysis.

(E-F) ES-2 cells were transfected with siRNAs as indicated and treated with DMSO or cisplatin as indicated for 48 h, followed by GSH assay.

(G and H) ES-2 cells were transfected with siRNAs as indicated and treated with DMSO or cisplatin as indicated for 24 h, followed by colony formation assay.

Data are represented as mean ± SD, $n = 3$. *$p < 0.05$, **$p < 0.01$.

Figure S13. Co-targeting mutant p53-R248Q and SNRPD2 via iRGD-decorated exosomes suppresses ovarian cancer. Related to Figure 6.

(A) Schematic representation of the treatment timeline for the mouse model.

(B-E) The growth rate (B), and weight (C), and size (D) of xenograft tumors derived from OVCAR-3 cells, along with mouse weight (E), were analyzed as described in the methods section.

Data in (B, C, and E) are presented as the mean ± SD, n = 6. **$p < 0.01$, ns, not significant.

Figure S14. SNRPD2 is upregulated in ovarian cancer and amplified in most cancers, related to poor prognosis in ovarian cancer patients. Related to Figure 7.

(A and B) SNRPD2 is highly expressed in multiple malignancies. Data were obtained from

The Cancer Genome Atlas (TCGA) and the Genotype-Tissue Expression Project (GTEx) using Sangerbox (https://www.sangerbox.com).

(C) The *SNRPD2* gene is amplified in malignancies. Data were obtained from TCGA and GTEx and analyzed via cBioPortal (http://www.cbioportal.org).

(D) Higher SNRPD2 expression is significantly associated with poor prognosis in ovarian cancer patients. Data were obtained from the Kaplan-Meier Plotter (https://kmplot.com).

Figure S1

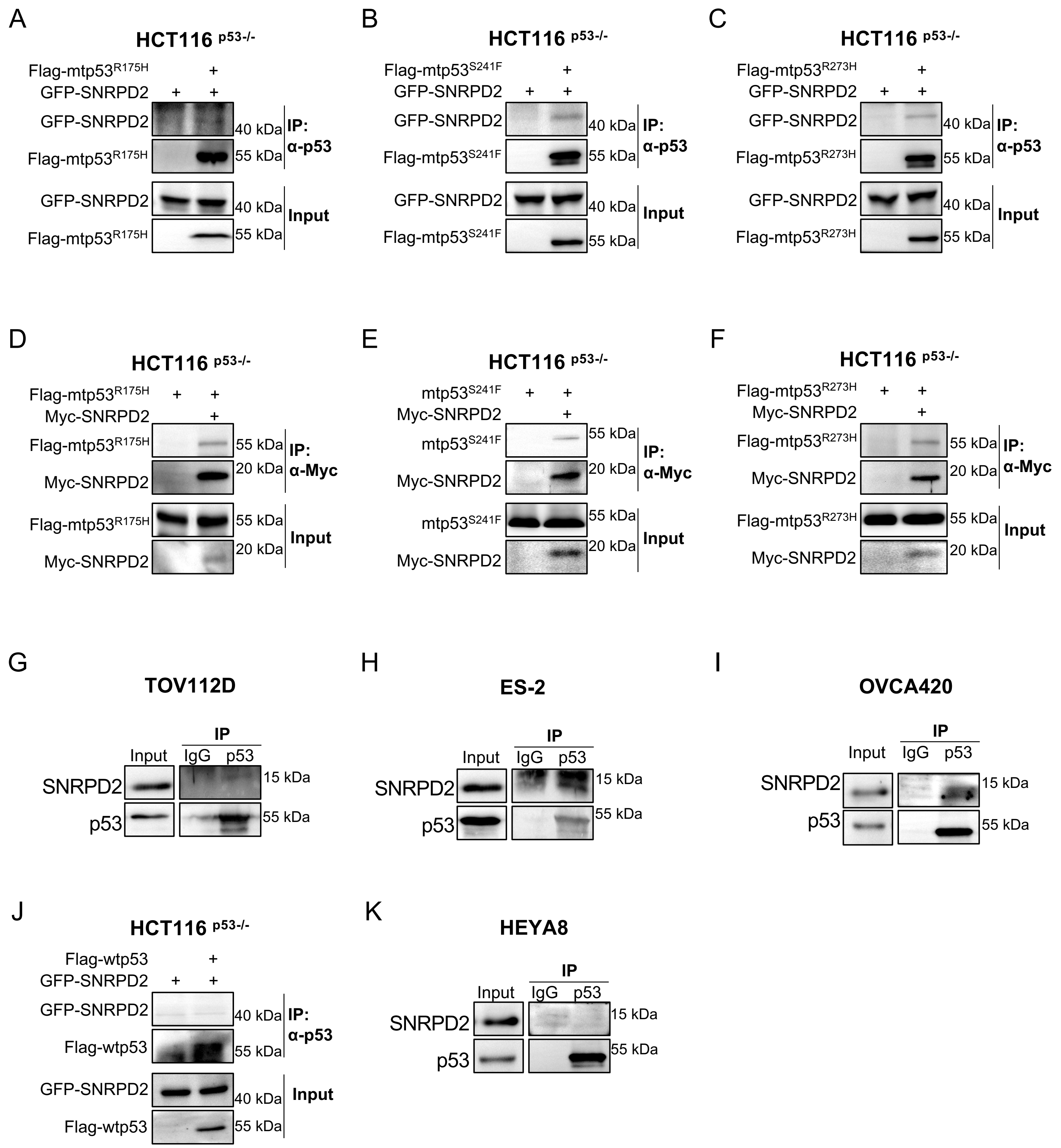
A
HCT116 p53-/-
Flag-mtp53R175H
GFP-SNRPD2
GFP-SNRPD2
Flag-mtp53R175H
40 kDa
55 kDa
IP: α-p53
Input
B
HCT116 p53-/-
Flag-mtp53S241F
GFP-SNRPD2
Flag-mtp53S241F
IP: α-p53
Input
C
HCT116 p53-/-
Flag-mtp53R273H
GFP-SNRPD2
Flag-mtp53R273H
IP: α-p53
Input
D
HCT116 p53-/-
Flag-mtp53R175H
Myc-SNRPD2
20 kDa
IP: α-Myc
Input
E
HCT116 p53-/-
mtp53S241F
Myc-SNRPD2
IP: α-Myc
Input
F
HCT116 p53-/-
Flag-mtp53R273H
Myc-SNRPD2
IP: α-Myc
Input
G
TOV112D
H
ES-2
I
OVCA420
IP
Input
IgG
p53
SNRPD2
15 kDa
J
HCT116 p53-/-
Flag-wtp53
GFP-SNRPD2
IP: α-p53
Input
K
HEYA8

Figure S2

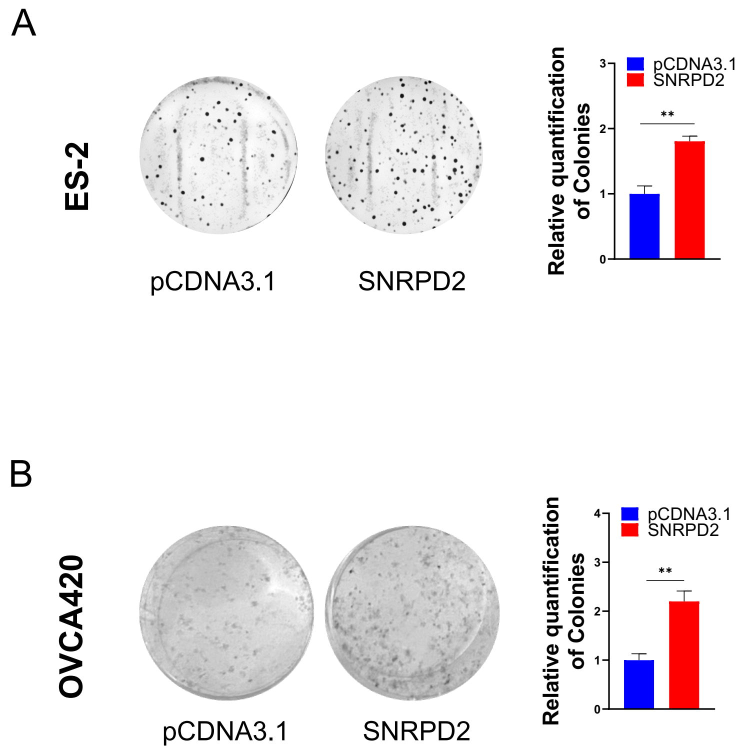
A
ES-2
pCDNA3.1
SNRPD2
Relative quantification of Colonies
pCDNA3.1
SNRPD2
**
B
OVCA420
pCDNA3.1
SNRPD2
Relative quantification of Colonies
pCDNA3.1
SNRPD2
**

Figure S3

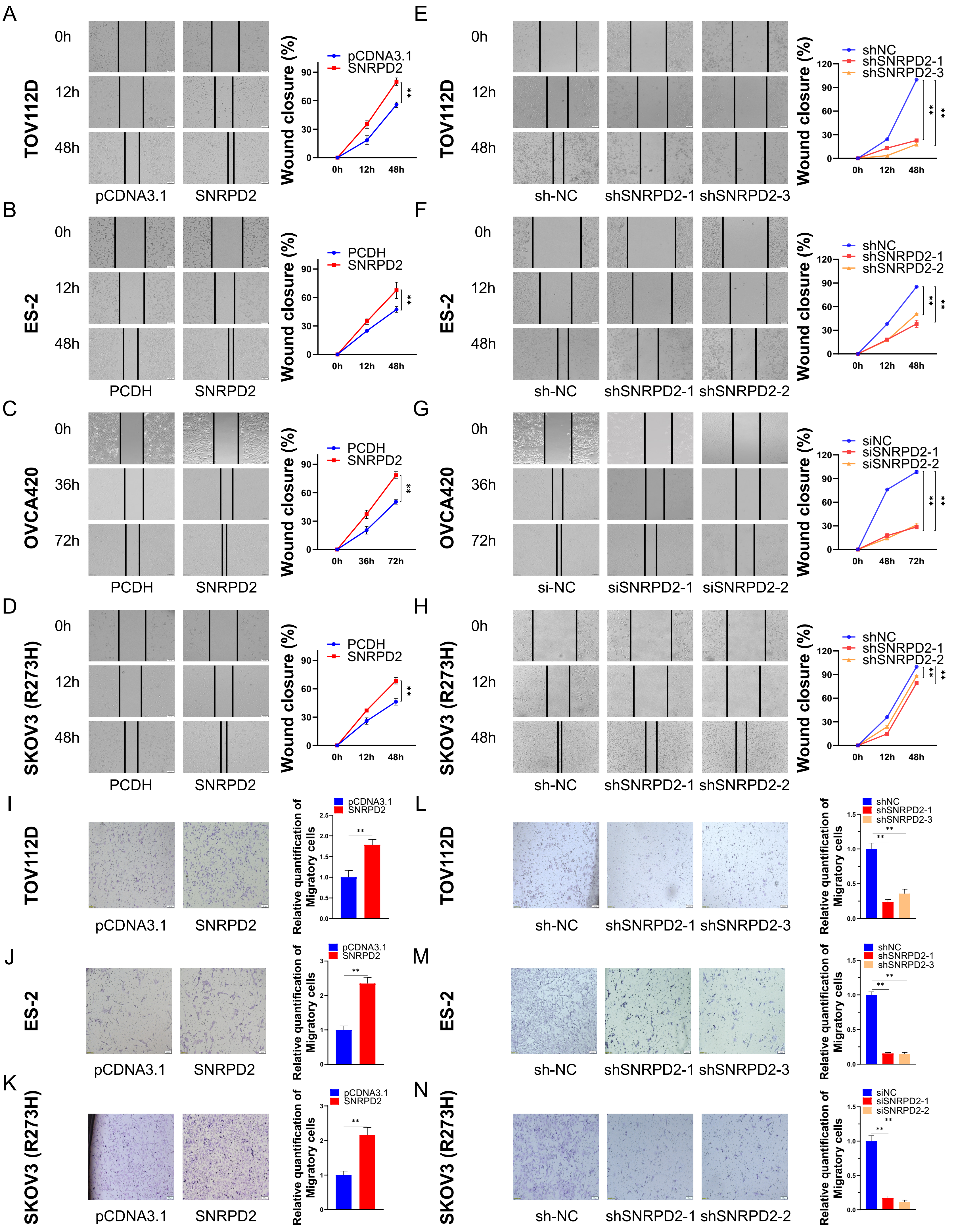
A
B
C
D
E
F
G
H
I
J
K
L
M
N
TOV112D
ES-2
OVCA420
SKOV3 (R273H)
0h
12h
36h
48h
72h
pCDNA3.1
PCDH
SNRPD2
sh-NC
si-NC
shSNRPD2-1
shSNRPD2-2
shSNRPD2-3
siSNRPD2-1
siSNRPD2-2
shNC
siNC
Wound closure (%)
Relative quantification of Migratory cells
**

Figure S4

A

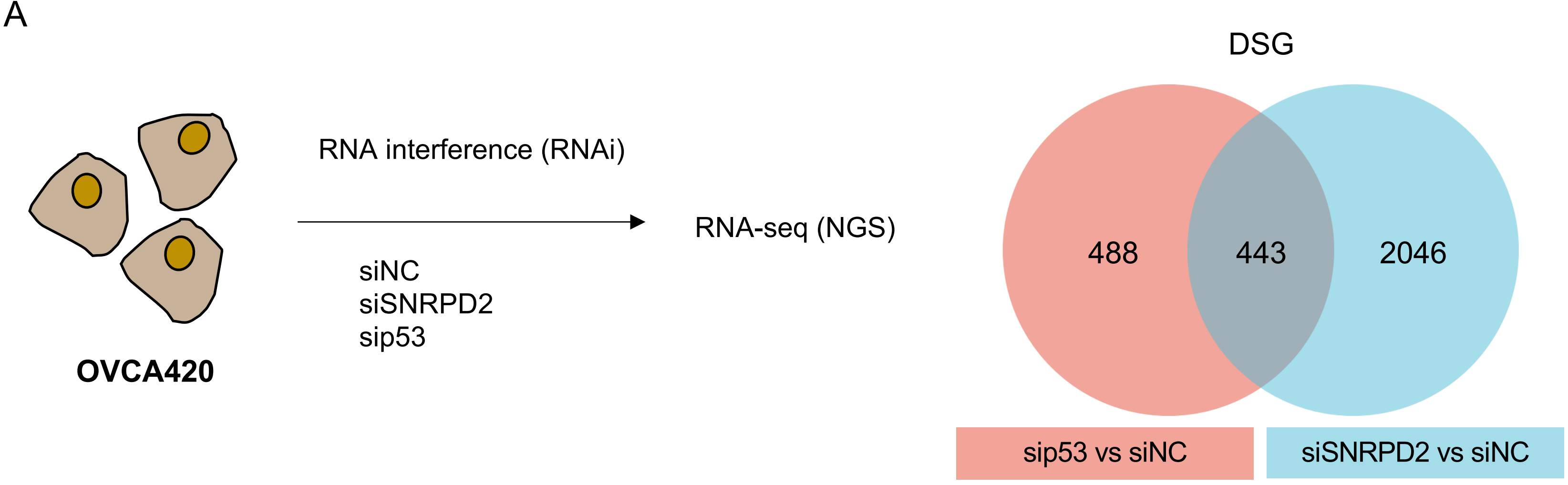
RNA interference (RNAi)
RNA-seq (NGS)
siNC
siSNRPD2
sip53
OVCA420
DSG
488
443
2046
sip53 vs siNC
siSNRPD2 vs siNC

B

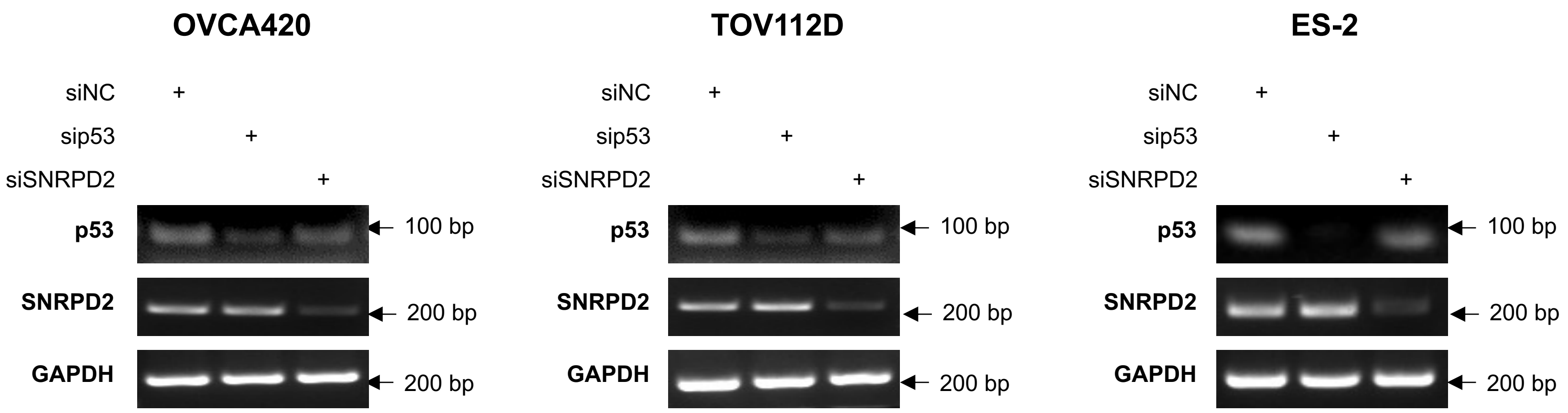
OVCA420
TOV112D
ES-2
siNC
sip53
siSNRPD2
p53
SNRPD2
GAPDH
100 bp
200 bp
200 bp

Figure S5

A

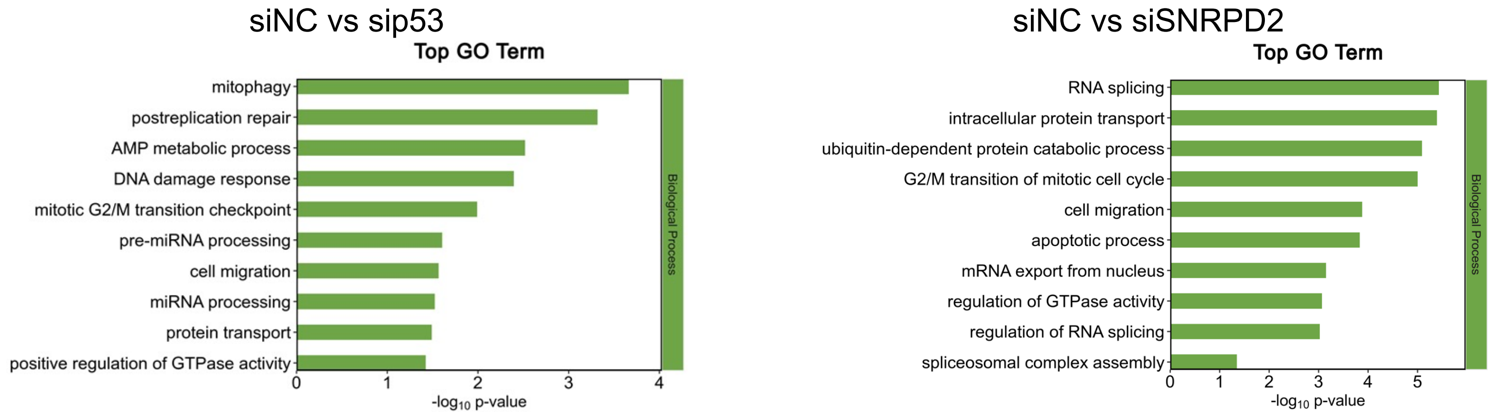

B

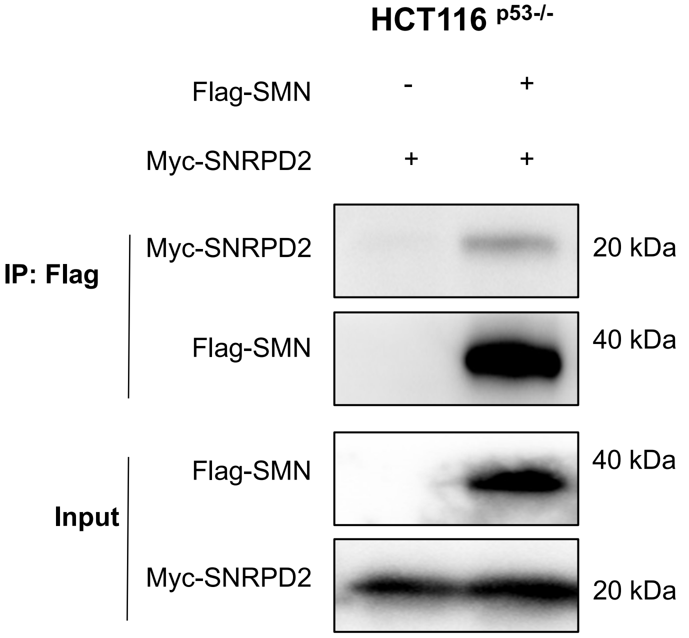

C

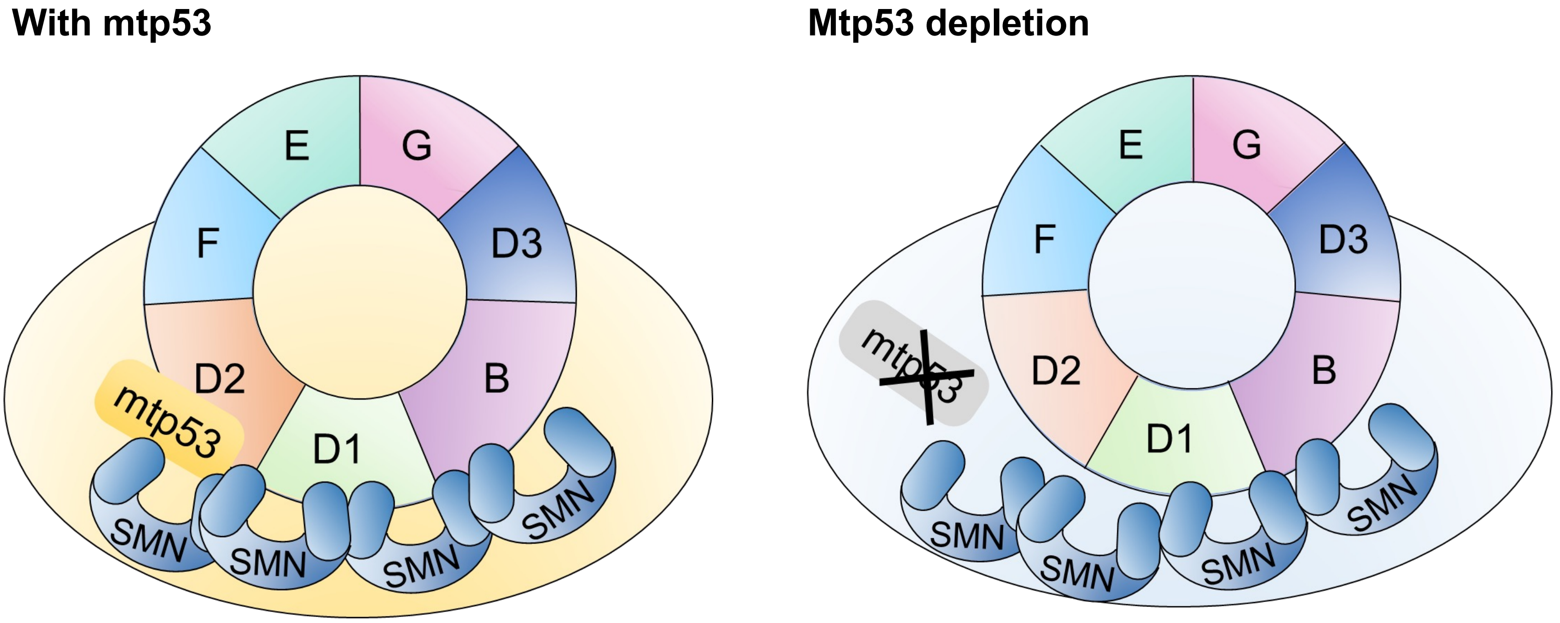

Figure S6

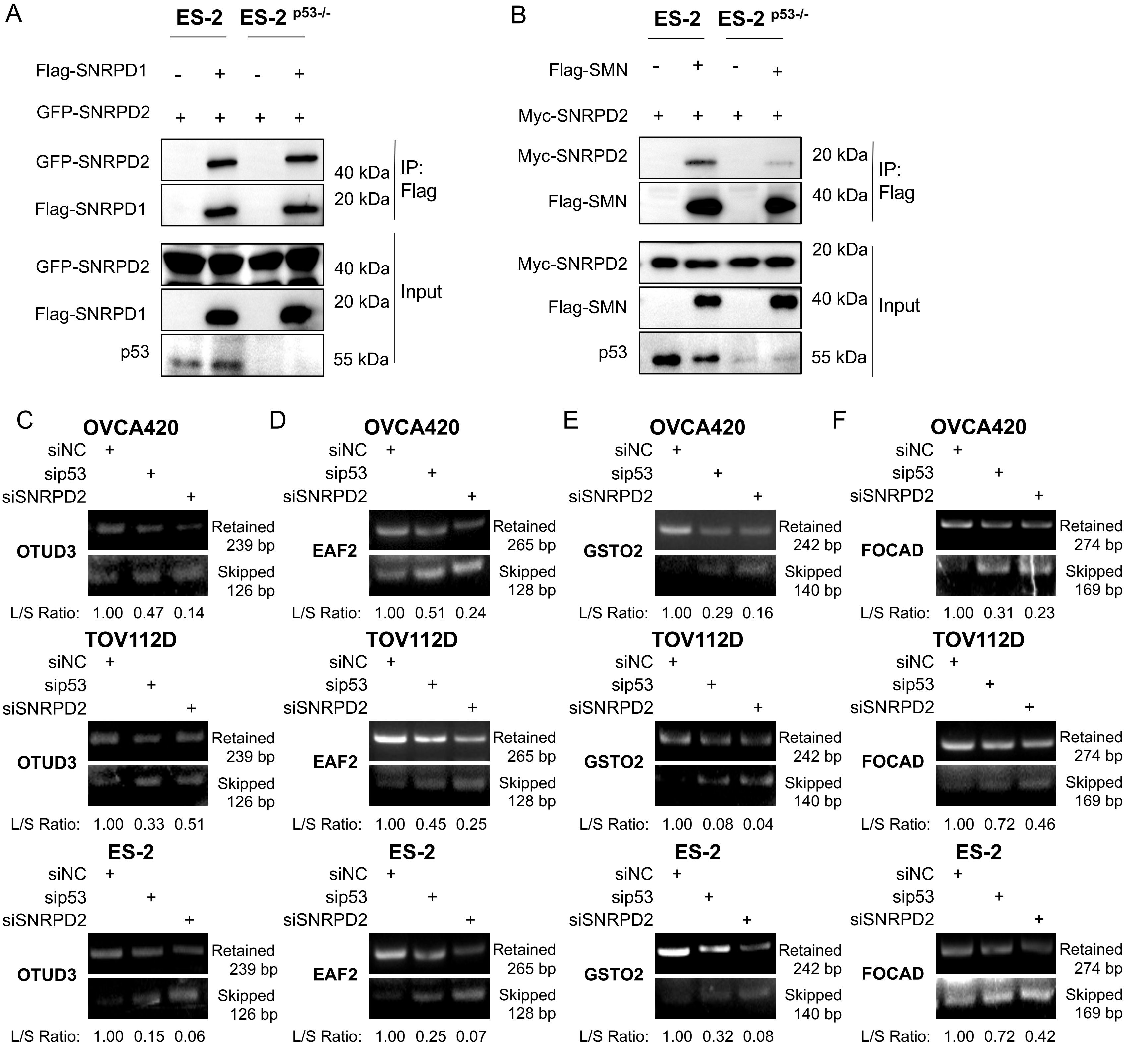
A
ES-2
ES-2 p53-/-
Flag-SNRPD1 - + - +
GFP-SNRPD2 + + + +
GFP-SNRPD2
Flag-SNRPD1
40 kDa
20 kDa
IP: Flag
GFP-SNRPD2
Flag-SNRPD1
p53
40 kDa
20 kDa
55 kDa
Input
B
ES-2
ES-2 p53-/-
Flag-SMN - + - +
Myc-SNRPD2 + + + +
Myc-SNRPD2
Flag-SMN
20 kDa
40 kDa
IP: Flag
Myc-SNRPD2
Flag-SMN
p53
20 kDa
40 kDa
55 kDa
Input
C
OVCA420
siNC
sip53
siSNRPD2
OTUD3
Retained 239 bp
Skipped 126 bp
L/S Ratio: 1.00 0.47 0.14
TOV112D
OTUD3
L/S Ratio: 1.00 0.33 0.51
ES-2
OTUD3
L/S Ratio: 1.00 0.15 0.06
D
OVCA420
EAF2
Retained 265 bp
Skipped 128 bp
L/S Ratio: 1.00 0.51 0.24
TOV112D
L/S Ratio: 1.00 0.45 0.25
ES-2
L/S Ratio: 1.00 0.25 0.07
E
OVCA420
GSTO2
Retained 242 bp
Skipped 140 bp
L/S Ratio: 1.00 0.29 0.16
TOV112D
L/S Ratio: 1.00 0.08 0.04
ES-2
L/S Ratio: 1.00 0.32 0.08
F
OVCA420
FOCAD
Retained 274 bp
Skipped 169 bp
L/S Ratio: 1.00 0.31 0.23
TOV112D
L/S Ratio: 1.00 0.72 0.46
ES-2
L/S Ratio: 1.00 0.72 0.42

Figure S7

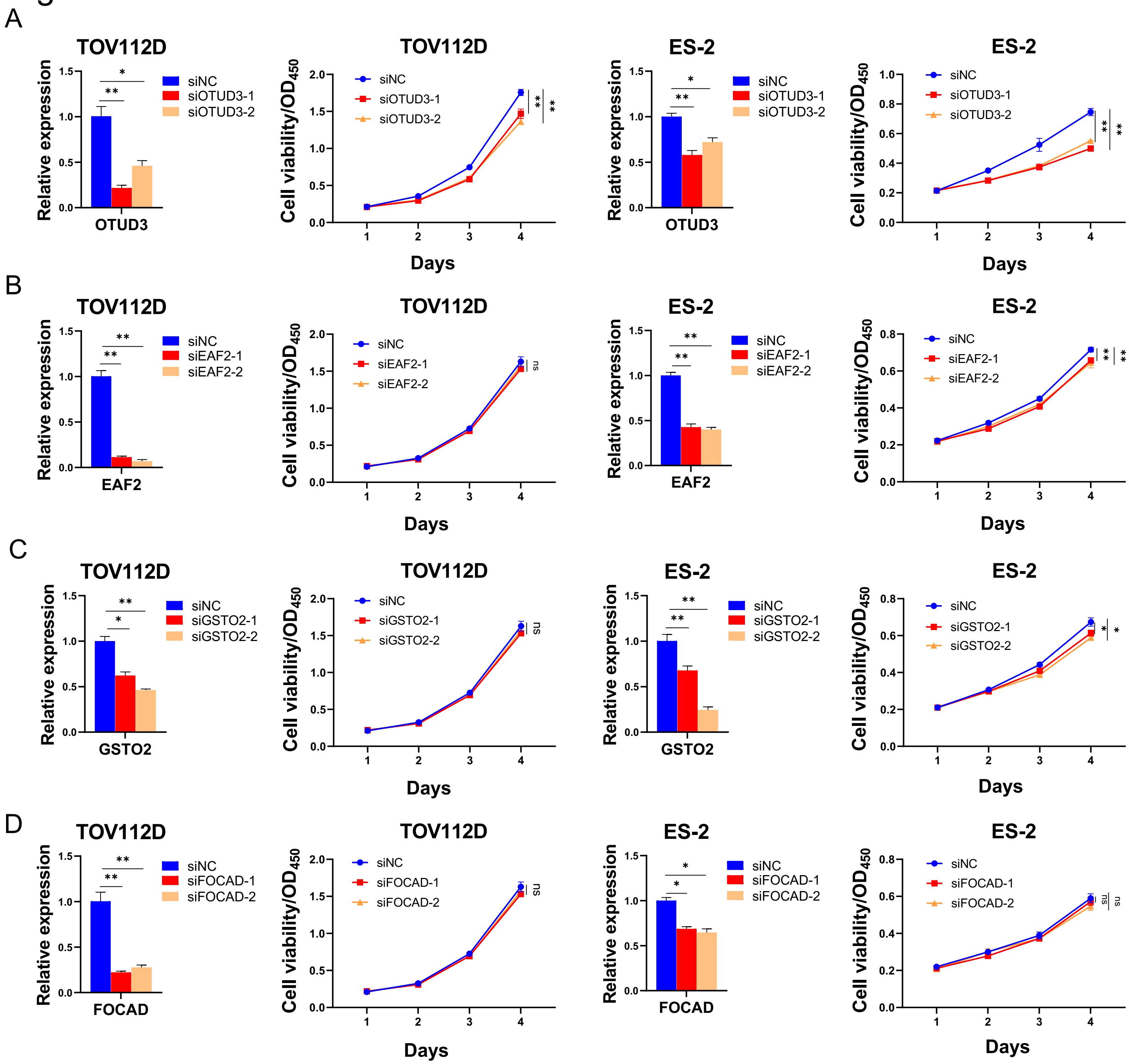
A
TOV112D
Relative expression
OTUD3
siNC
siOTUD3-1
siOTUD3-2
Cell viability/OD450
Days
ES-2
B
EAF2
siEAF2-1
siEAF2-2
C
GSTO2
siGSTO2-1
siGSTO2-2
D
FOCAD
siFOCAD-1
siFOCAD-2
ns

Figure S8

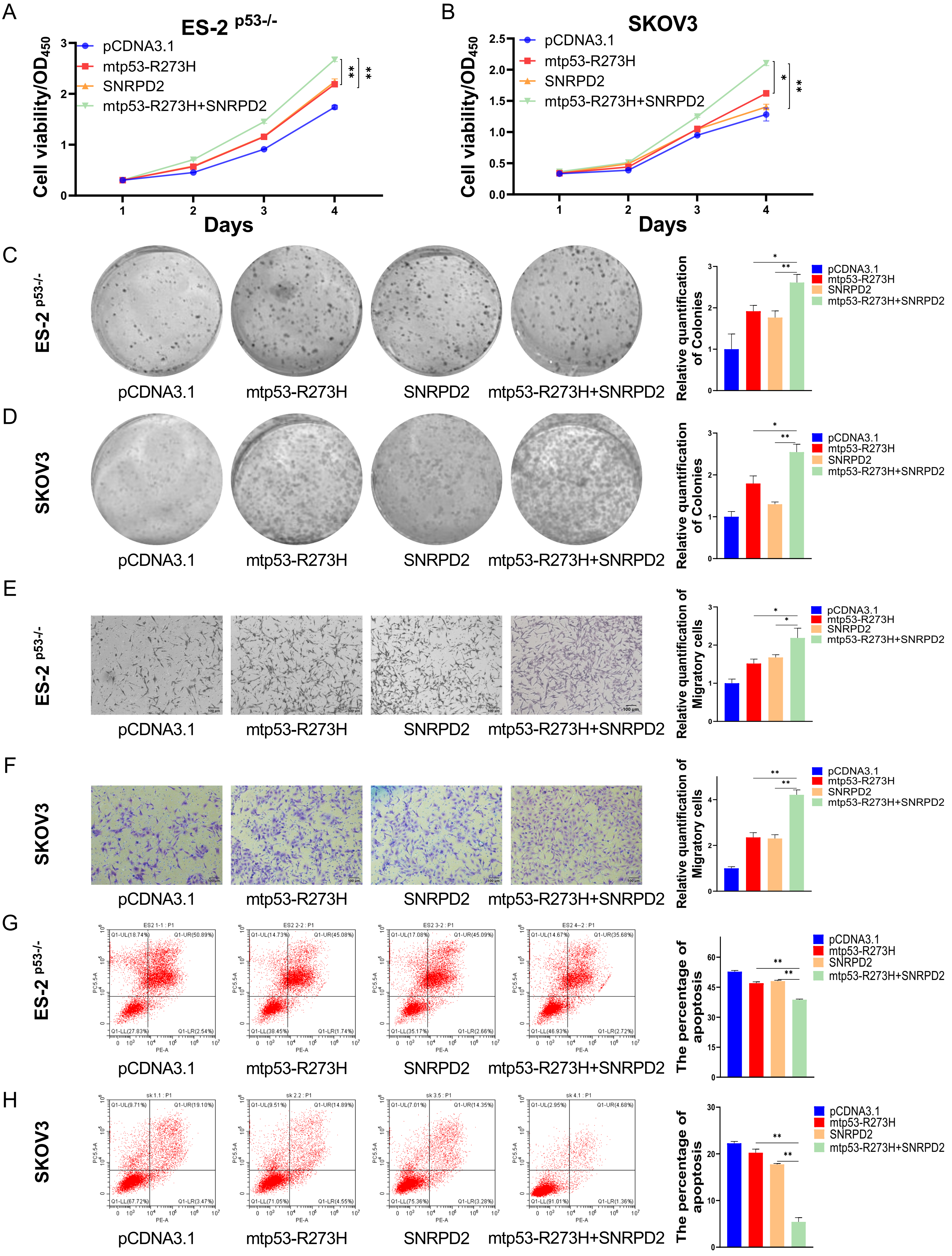
A
ES-2 p53-/-
Cell viability/OD450
Days
pCDNA3.1
mtp53-R273H
SNRPD2
mtp53-R273H+SNRPD2
B
SKOV3
C
ES-2 p53-/-
Relative quantification of Colonies
D
SKOV3
E
Relative quantification of Migratory cells
F
G
The percentage of apoptosis
H

Figure S9

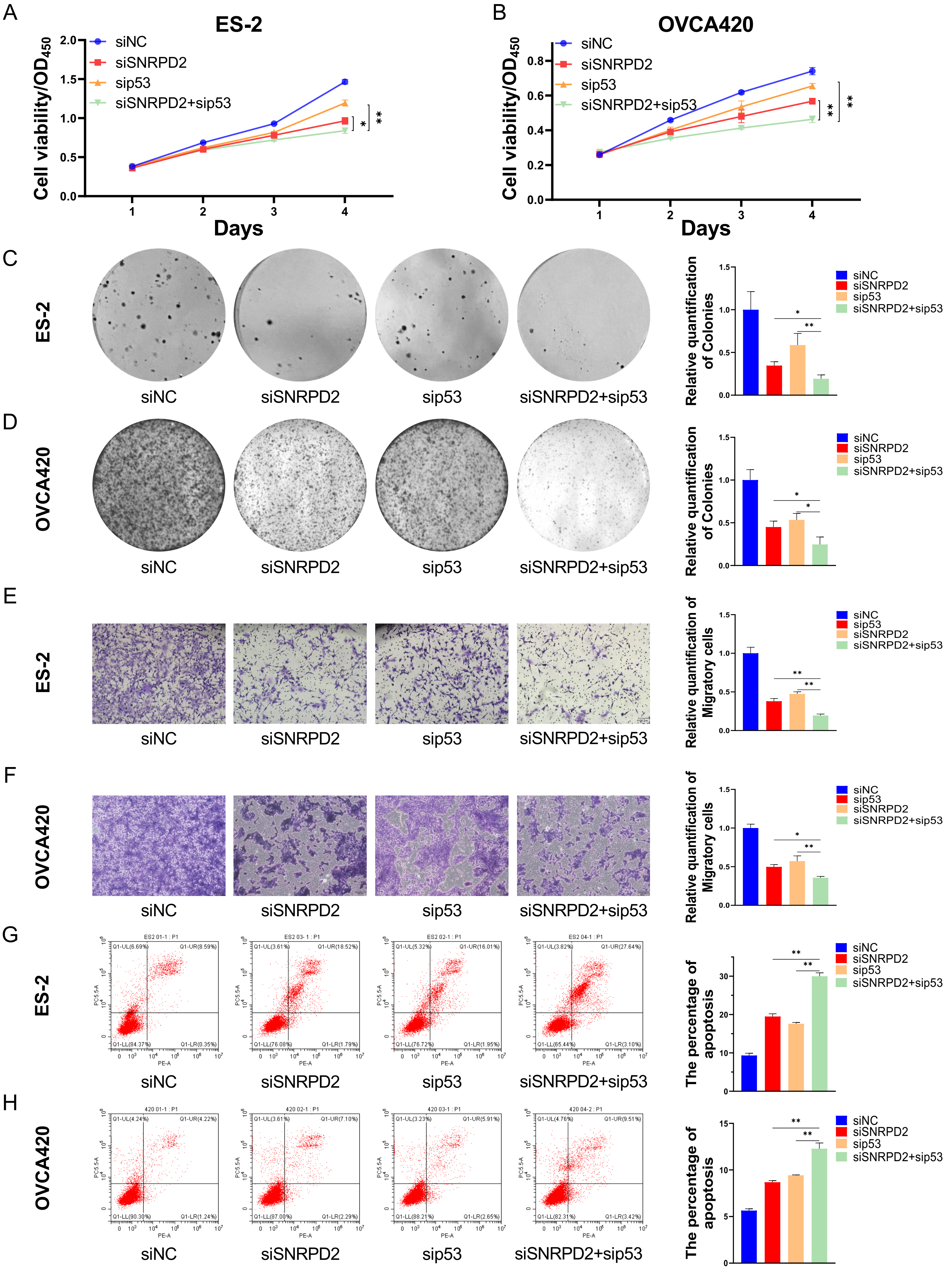
A
ES-2
Cell viability/OD450
siNC
siSNRPD2
sip53
siSNRPD2+sip53
Days
B
OVCA420
C
ES-2
siNC
siSNRPD2
sip53
siSNRPD2+sip53
Relative quantification of Colonies
D
OVCA420
E
Relative quantification of Migratory cells
F
G
The percentage of apoptosis
H

Figure S10

A

p53 WT ------- TGCATGGGCGGCATGAACCGGAGGCCCATCCTCACCATCATCACACT

p53 MUT-R248Q ------- TGCATGGGCGGCATGAACCAGAGGCCCATCCTCACCATCATCACACT

sip53-2 ------- CGGCAUGAACCGGAGGCCCAU

siR248Q-1 ------- GCGGCAUGAACAAGAGGCCC

siR248Q-2 ------- GCGGCAUGAAACAGAGGCCC

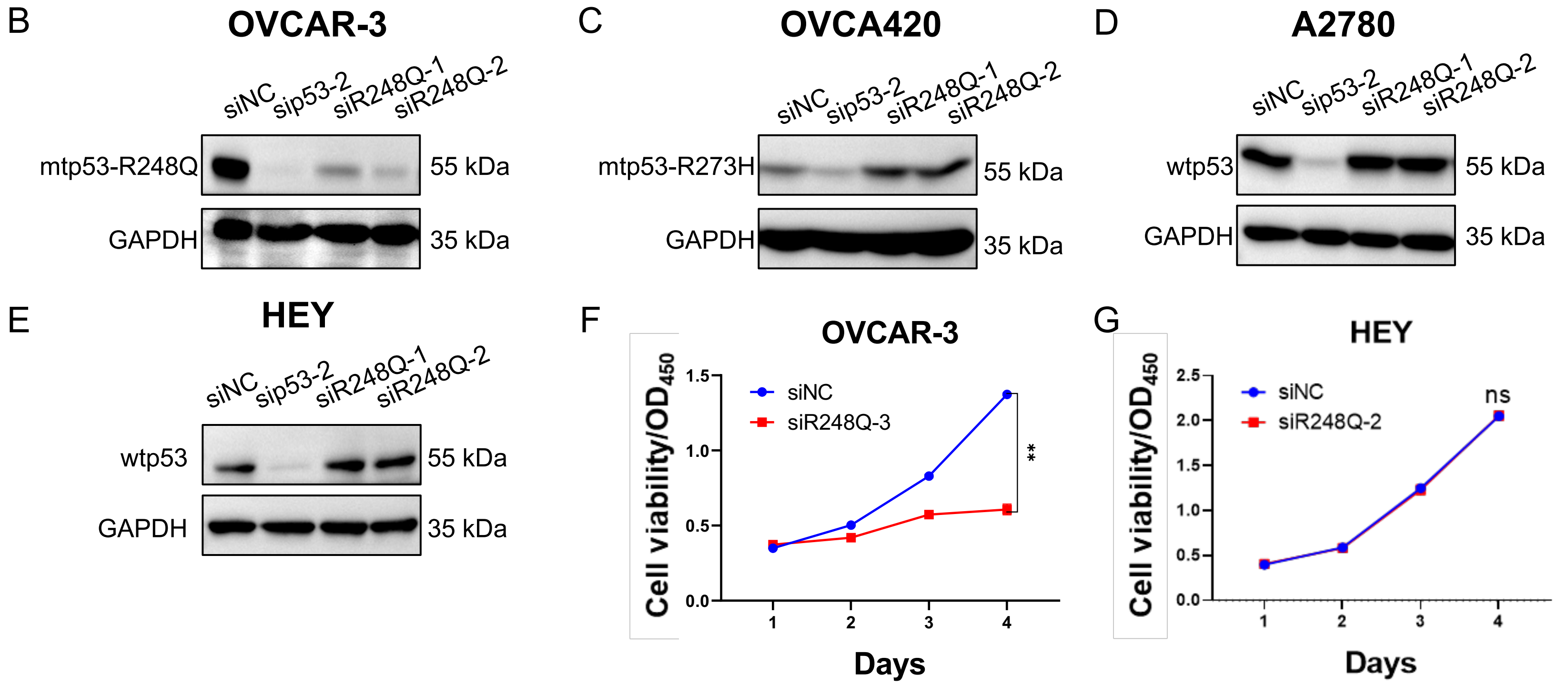

Figure S11

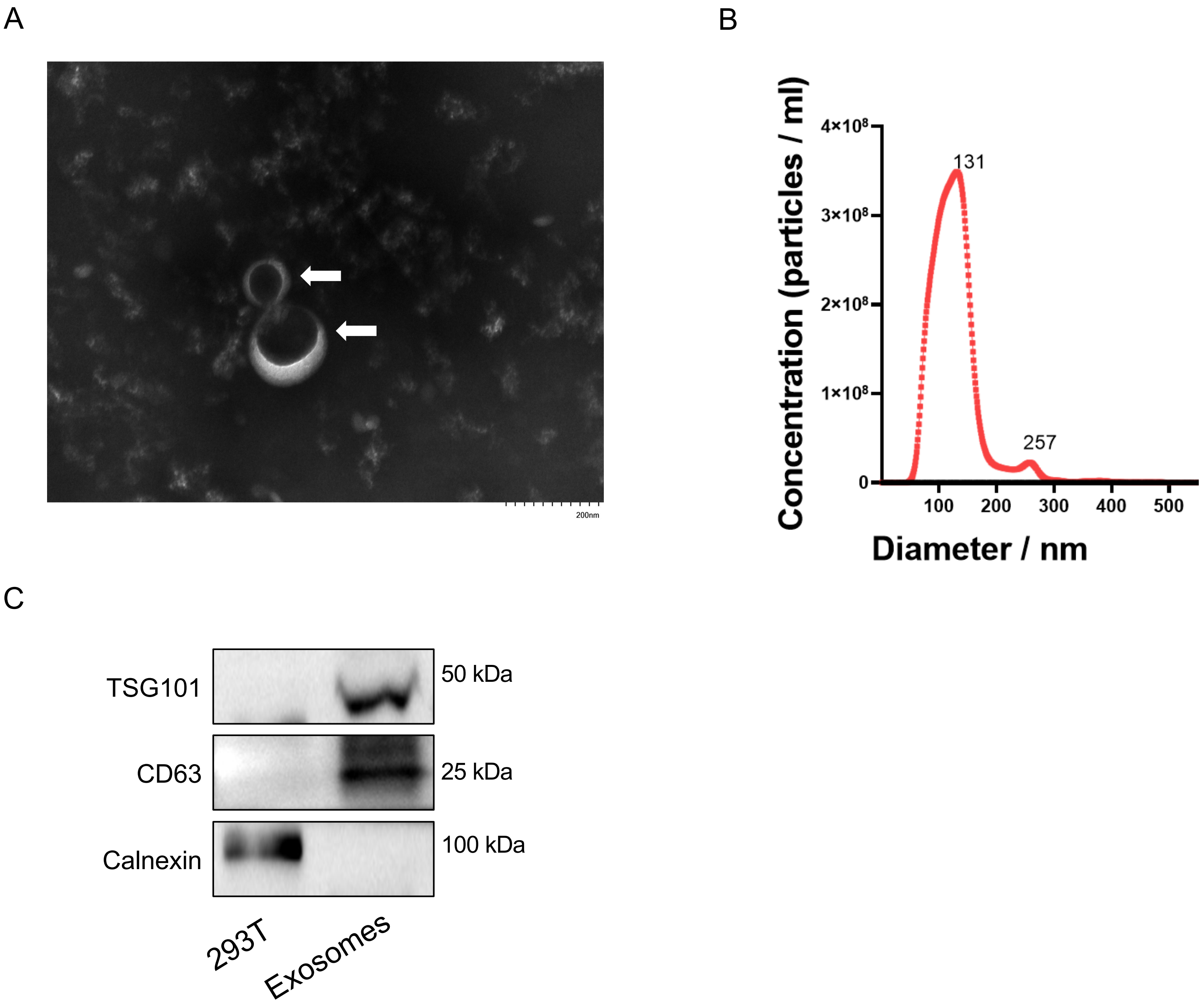
A
200nm
B
Concentration (particles / ml)
4×10⁸
3×10⁸
2×10⁸
1×10⁸
0
131
257
100
200
300
400
500
Diameter / nm
C
TSG101
CD63
Calnexin
50 kDa
25 kDa
100 kDa
293T
Exosomes

Figure S12

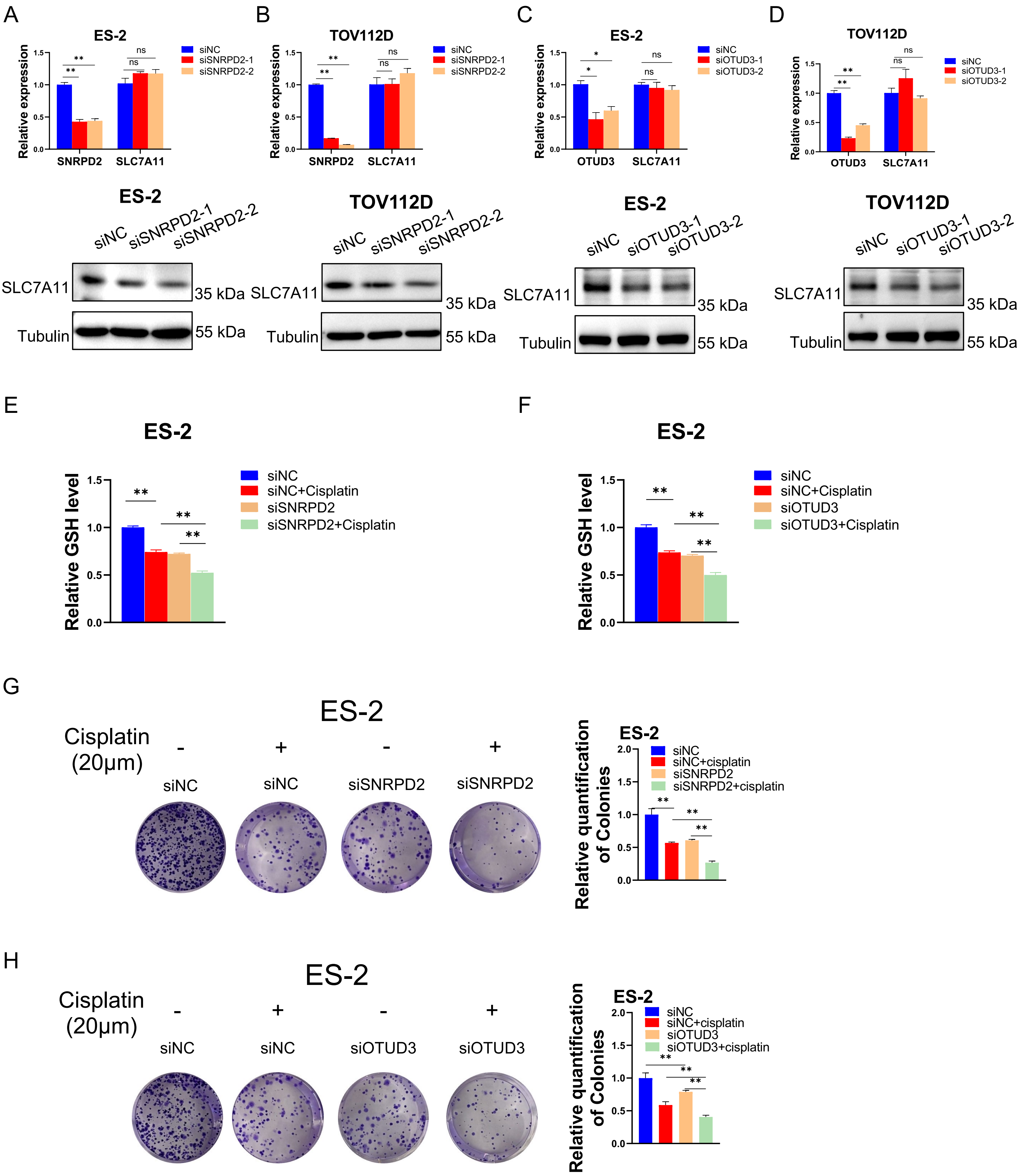
A
ES-2
Relative expression
SNRPD2
SLC7A11
siNC
siSNRPD2-1
siSNRPD2-2
B
TOV112D
C
ES-2
OTUD3
siOTUD3-1
siOTUD3-2
D
TOV112D
ES-2
siNC siSNRPD2-1 siSNRPD2-2
SLC7A11
35 kDa
Tubulin
55 kDa
TOV112D
siNC siOTUD3-1 siOTUD3-2
E
ES-2
Relative GSH level
siNC
siNC+Cisplatin
siSNRPD2
siSNRPD2+Cisplatin
F
ES-2
siOTUD3
siOTUD3+Cisplatin
G
ES-2
Cisplatin (20μm)
- + - +
siNC siNC siSNRPD2 siSNRPD2
Relative quantification of Colonies
siNC+cisplatin
siSNRPD2+cisplatin
H
ES-2
siNC siNC siOTUD3 siOTUD3
siOTUD3+cisplatin

Figure S13

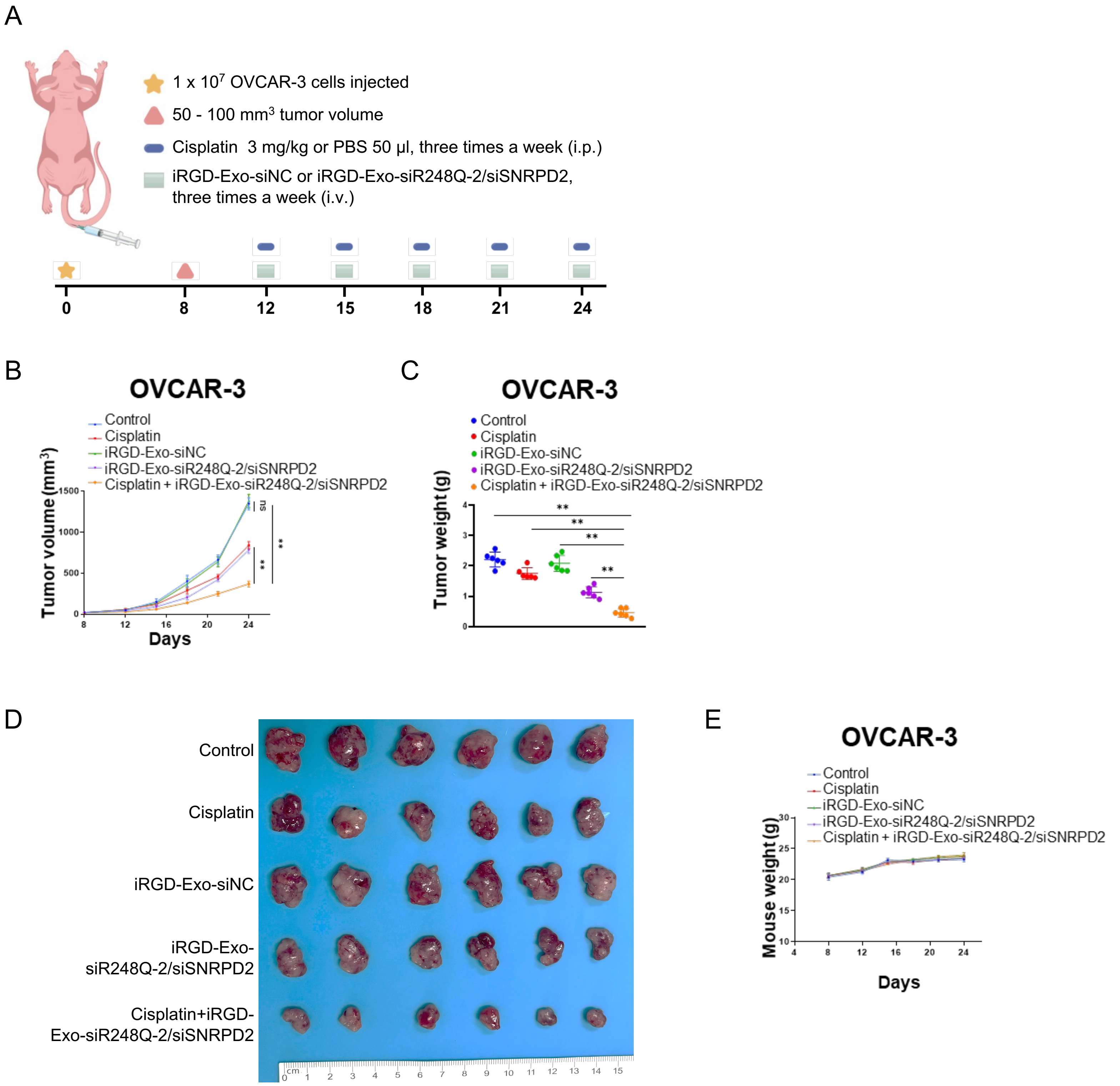

A
1 x 10^7 OVCAR-3 cells injected
50 - 100 mm^3 tumor volume
Cisplatin 3 mg/kg or PBS 50 µl, three times a week (i.p.)
iRGD-Exo-siNC or iRGD-Exo-siR248Q-2/siSNRPD2, three times a week (i.v.)
0
8
12
15
18
21
24
B
OVCAR-3
Control
Cisplatin
iRGD-Exo-siNC
iRGD-Exo-siR248Q-2/siSNRPD2
Cisplatin + iRGD-Exo-siR248Q-2/siSNRPD2
Tumor volume (mm^3)
Days
ns
**
**
C
OVCAR-3
Control
Cisplatin
iRGD-Exo-siNC
iRGD-Exo-siR248Q-2/siSNRPD2
Cisplatin + iRGD-Exo-siR248Q-2/siSNRPD2
Tumor weight (g)
**
D
Control
Cisplatin
iRGD-Exo-siNC
iRGD-Exo-siR248Q-2/siSNRPD2
Cisplatin+iRGD-Exo-siR248Q-2/siSNRPD2
E
OVCAR-3
Control
Cisplatin
iRGD-Exo-siNC
iRGD-Exo-siR248Q-2/siSNRPD2
Cisplatin + iRGD-Exo-siR248Q-2/siSNRPD2
Mouse weight (g)
Days

Figure S14

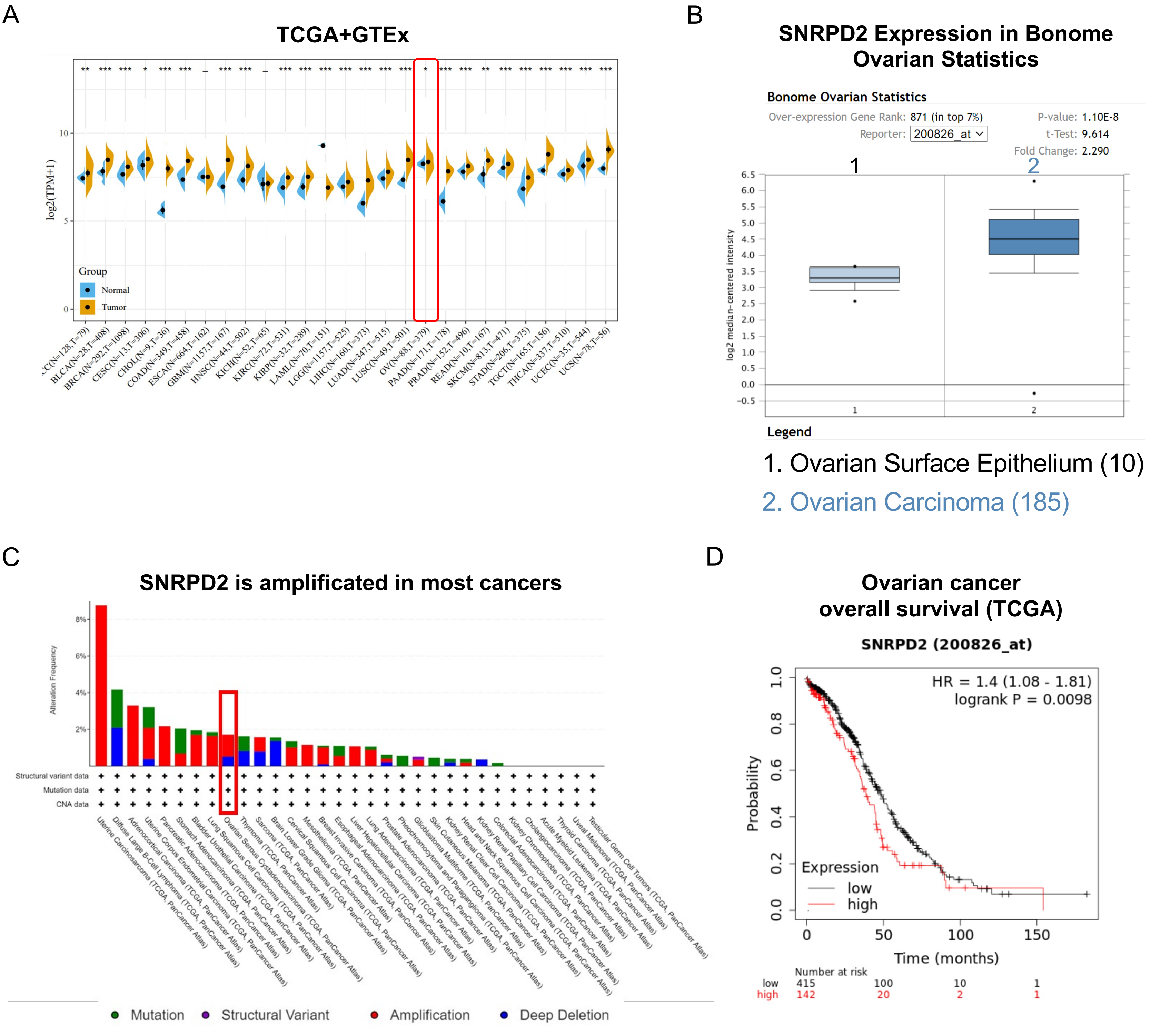
A
TCGA+GTEx
log2(TPM+1)
Group
Normal
Tumor
B
SNRPD2 Expression in Bonome Ovarian Statistics
Bonome Ovarian Statistics
Over-expression Gene Rank: 871 (in top 7%)
Reporter: 200826_at
P-value: 1.10E-8
t-Test: 9.614
Fold Change: 2.290
log2 median-centered intensity
Legend
1. Ovarian Surface Epithelium (10)
2. Ovarian Carcinoma (185)
C
SNRPD2 is amplificated in most cancers
Alteration Frequency
Structural variant data
Mutation data
CNA data
Mutation
Structural Variant
Amplification
Deep Deletion
D
Ovarian cancer overall survival (TCGA)
SNRPD2 (200826_at)
HR = 1.4 (1.08 - 1.81)
logrank P = 0.0098
Probability
Expression
low
high
Time (months)
Number at risk
low 415 100 10 1
high 142 20 2 1

Supplementary tables

Table S1. Relationship between SNRPD2 expression and clinicopathologic factors of patients with HGSOC

Characteristic	HGSOC Patients, N (%)		*p*-value
	Low-SNRPD2 N=10(13.5%)	High-SNRPD2 N=64(86.5%)	
**Age (years)**			0.41
≤60	6(60.0%)	49(76.6%)	
>60	4(40.0%)	15(23.4%)	
**FIGO stage**			**0.03**
I	0(0.0%)	8(12.5%)	
II	0(0.0%)	4(6.3%)	
III	5(50.0%)	32(50.0%)	
IV	5(50.0%)	20(31.2%)	
**Residual disease**			0.17
R0	2(20.0%)	36(56.3%)	
R1	5(50.0%)	18(28.1%)	
>R1	3(30.0%)	10(15.6%)	

p values by χ^2 test or Fisher's exact test.

Table S2. Primers for plasmid construction

Myc pcDNA3.1-EcoRI-SNRPD2-F	5'-CGGAATTCATGAGCCTCCTCAACAAGCC-3'
Myc pcDNA3.1-BamHI-SNRPD2-R	5'-CGGGATCCCTTGCCGGCGATGAGC-3'
GFP pWPXL-BamHI-SNRPD2-F	5'-CGGGATCCATGAGCCTCCTCAACAA-3'
GFP pWPXL-MIU-SNRPD2-R	5'-CGACGCGTCTCTTGCCGGCGATGA-3'
Flag PCDH-BamHI-OTUD3-F	5'-ATTCGAATTTAAATCGGATCCATGTCCCGAAAGCAGGCG-3'
Flag PCDH-XhOI-OTUD3-R	5'-GCACAGTCGCTGCAGCTCGAGTCAGATGTTGAGAGCGGCG-3'

Table S3. Primers for shRNA construction

shSNRPD2-1-F	5’-CCGGCATCAACTGCCGCAACAATAACTCGAGTTATTGTT GCGGCAGTTGATGTTTTTG-3’
shSNRPD2-1-R	5’-AATTCAAAAACATCAACTGCCGCAACAATAACTCGAGTT ATTGTTGCGGCAGTTGATG-3’
shSNRPD2-2-F	5’-CCGGGGAGATGTGGACTGAGGTACTCGAGTACCTCAGTC CACATCTCCTTTTTG-3’
shSNRPD2-2-R	5’-AATTCAAAAAGGAGATGTGGACTGAGGTACTCGAGTACC TCAGTCCACATCTCC-3’
shSNRPD2-3-F	5’-CCGGCGATAGGCACTGCAACATGGTCTCGAGACCATGTT GCAGTGCCTATCGTTTTTG-3’
shSNRPD2-3-R	5’-AATTCAAAAACGATAGGCACTGCAACATGGTCTCGAGAC CATGTTGCAGTGCCTATCG-3’

Table S4. Sequences for siRNAs

siNC	5’-UUCUCCGAACGUGUCACGU-3’
siSNRPD2-1	5’-GGAAUUUAACACCGGUCCA-3’
siSNRPD2-2	5’-GGAGAUGUGGACUGAGGUA-3’
siOTUD3-1	5’-GGGAAGAUUUUGAACCCUU-3’
siOTUD3-2	5’-GUGGCAGUGGUGCCAGAAU-3’
sip53-1	5’-AACUACAUGUGUAACAGUUCCUTT-3’
siS241F-1	5’-AACUACAUGUGUAACAGUUUCUTT-3’
siS241F-2	5’-UUUCUGCAUGGGCGGCAUGTT-3’
siS241F-3	5’-AACUACAUGUGUAACAGUGUCUTT-3’
siS241F-4	5’-AACUACAUGUGUAACAGGUUCUTT-3’
siEAF2-1	5’-GGAUACCUUGAGGUUGGUGTT-3’
siEAF2-2	5’-GGCAGAAGCUAGUCUAAUGTT-3’
siGSTO2-1	5’-GCUGUUUCCAUAUGACCCUTT-3’
siGSTO2-2	5’-CCUGUAUAUCCAUGAUUGATT-3’
siFOCAD-1	5’-GGCGAUGAUGUUUAUUGAGTT-3’
siFOCAD-2	5’-GCAUCAAUCAGAGAUAUAUTT-3’
sip53-2	5’-CGGCAUGAACCGGAGGCCCAU-3’
siR248Q-1	5’-GCGGCAUGAACAAGAGGCCC-3’
siR248Q-2	5’-GCGGCAUGAAACAGAGGCCC-3’

Table S5. Primers for quantitative PCR

FOCAD-F	5’-AACAGCCTGCTGTGAAGGTC-3’
FOCAD-R	5’-AGCTGCTGCAAAAACACTGG-3’
GSTO2-F	5’-TGAACGAGCTCGCCAAAAGA-3’
GSTO2-R	5’-CATACACATCCAGCCGCTCA-3’
OTUD3-F	5’-GCACAGACAGGAGACAGTGG-3’
OTUD3-R	5’-GTAACTCCCTCACGCTGCTT-3’
EAF2-F	5’-AGGTTGGTGAAGGTGAACAGG-3’
EAF2-R	5’-AGATTGGGAGTCCTGGCTGA-3’
GAPDH-F	5’-GGAGCGAGATCCCTCCAAAAT-3’
GAPDH-R	5’-GGCTGTTGTCATACTTCTCATGG-3’
P53-F	5’-CCCAAGCAATGGATGATTTGA-3’
P53-R	5’-GGCATTCTGGGAGCTTCATCT-3’
SNRPD2-F	5’-CCTCCTCAACAAGCCCAAGA-3’
SNRPD2-R	5’-CTTCACGTTCTCCAGCACCA-3’
